# Spatiotemporal kinetics of the SRP pathway in live *E. coli* cells

**DOI:** 10.1073/pnas.2204038119

**Published:** 2022-09-12

**Authors:** Ivan L. Volkov, Erik Lundin, Kalle Kipper, Mikhail Metelev, Spartak Zikrin, Magnus Johansson

**Affiliations:** ^a^Department of Cell and Molecular Biology, Uppsala University, 752 36 Uppsala, Sweden

**Keywords:** translation, cotranslational targeting, single-molecule tracking, super-resolution microscopy, protein synthesis

## Abstract

Mechanistic details of the signal recognition particle (SRP)-mediated insertion of membrane proteins have been described from decades of in vitro biochemical studies. However, the dynamics of the pathway inside the living cell remain obscure. By combining in vivo single-molecule tracking with numerical modeling and simulated microscopy, we have constructed a quantitative reaction–diffusion model of the SRP cycle. Our results suggest that the SRP–ribosome complex finds its target, the membrane-bound translocon, through a combination of three-dimensional (3D) and 2D diffusional search, together taking on average 750 ms. During this time, the nascent peptide is expected to be elongated only 12 or 13 amino acids, which explains why, in *Escherichia coli*, no translation arrest is needed to prevent incorrect folding of the polypeptide in the cytosol. We also found that a remarkably high proportion (75%) of SRP bindings to ribosomes occur in the cytosol, suggesting that the majority of target ribosomes bind SRP before reaching the membrane. In combination with the average SRP cycling time, 2.2 s, this result further shows that the SRP pathway is capable of targeting all substrate ribosomes to translocons.

## Significance

Most of the proteins in all organisms are synthesized in the cell cytosol. However, a substantial fraction of these proteins have their function somewhere else, and cells therefore need protein targeting systems to relocate proteins during or after their synthesis. The signal recognition particle (SRP) is a universal player in protein targeting, but biochemical studies of its dynamics have been problematic since the geometric constraints inside living cells are hard to mimic in the test tube. Using single-molecule tracking, we have followed how SRP targets proteins to the membrane-bound translocation complexes, directly in living bacterial cells. Our kinetic measurements of the pathway will aid quantitative modeling and engineering of bacterial cells (e.g., for the production of medically relevant recombinant proteins).

The signal recognition particle (SRP) pathway is a universally conserved mechanism for cotranslational insertion or translocation of new proteins into or across the membrane. In bacteria, most or all inner-membrane proteins (IMPs), and a subset of periplasmic proteins, are believed to be targeted by the SRP pathway (1). In eukaryotes, SRP localizes nascent peptides cotranslationally to the endoplasmic reticulum (2). SRP binds at the nascent peptide exit tunnel of the large ribosomal subunit, where it recognizes a hydrophobic signal peptide in the N terminus of the nascent peptide and, with the assistance of the SRP receptor (SR) protein (FtsY in *Escherichia coli*) delivers the translating ribosome to the IMP translocation channel, the translocon. Upon correct insertion of the nascent peptide into the translocon, guanosine triphosphate (GTP) hydrolysis leads to dissociation of SRP and SR (3, 4), which are then ready for a new round of targeting. In *E. coli*, SRP consists of 4.5S RNA and the protein Ffh.

Biochemical studies of SRP in reconstituted systems, together with recent high-resolution structures of functional states, have helped in dissecting the SRP targeting pathway [reviewed in (3, 4)]. However, since cotranslational ribosome targeting to the translocons is a highly dynamic process, involving physical movement in a confined geometry very different from the test tube, a quantitative understanding of the process is lacking. For example, we have very sparse knowledge about the timing of the targeting events, and for the purpose of recombinant protein production, we do not know what the rate limiting steps are. Clearly, in *E. coli*, where SRP probably does not arrest translation elongation during targeting (1, 5), the search for a vacant translocon needs to be very efficient in order for the nascent peptide not to be incorrectly folded in the cytosol. How does this search occur? Is targeting to a translocon a pure three-dimensional (3D) diffusional search, or does it involve 2D diffusion in the membrane (6)? And do all potential SRP target ribosomes require SRP, or is it enough if the first ribosome on a messenger RNA (mRNA) is targeted to a translocon? Furthermore, whereas the traditional model of cotranslational targeting assumes that newly transcribed mRNAs are pulled to the membrane through SRP-mediated ribosome targeting to translocons, there are experimental results suggesting that IMP encoding mRNAs are membrane targeted independently of the SRP pathway (7), implying that SRP would target only ribosomes already localized by the membrane (8). But to what extent, if at all, this alternative pathway is exploited by the cell remains an open question.

To investigate the spatiotemporal kinetics of SRP-mediated ribosome targeting to the translocon directly inside the living cell, we have site-specifically dye-labeled the *E. coli* 4.5S RNA in vitro and then electroporated the molecules into living *E. coli* cells. By tracking the diffusion of 4.5S RNA at high temporal and spatial resolution, we were able to follow SRP through its reaction cycle and directly observe the SRP-assisted ribosome targeting to the translocon.

## Results

### Single-Molecule Tracking of 4.5S RNA.

Purified *E. coli* 4.5S RNA was labeled with the LD655 hydrazide dye through periodate oxidation of the 4.5S RNA 3′ terminus (9, 10). Based on available structural data (11, 12), and in vivo biochemical experiments (13, 14), dye-labeling at the 3′ end should not compromise the functionality of 4.5S RNA (see *SI Appendix, Supplementary Note 1*). Similar 3′ end-labeled 4.5S RNA has previously been assayed extensively in reconstituted systems, where no differences to the unlabeled RNA was discovered (15, 16). We found that all of the LD655-labeled 4.5S RNA binds Ffh in vitro (*SI Appendix*, Fig. S2).

The labeled 4.5S RNA was delivered into *E. coli* cells by electroporation. Electroporation of dye-labeled nucleic acids for single-molecule tracking was pioneered by the Kapanidis laboratory (17, 18), and has subsequently been used by us to deliver and track transfer RNA (tRNA) in living cells (19–22). After recovery, electroporated single cells were spread on a Rich Defined Medium (RDM) agarose pad, and data were subsequently acquired only from cells that, after incubation at 37 °C for 1.5 h, had formed small colonies via cell division (*SI Appendix*, Fig. S3). The agarose pad was, in addition, supplemented with SYTOX Blue dead cell stain, selectively highlighting dead cells with ruptured membrane, allowing us to discard dead cells from the subsequent analysis (*SI Appendix*, Fig. S3). Cells that survive electroporation, and their corresponding daughter cells, grow and divide normally before and after data acquisition (19) (*SI Appendix*, Figs. S3 and S4). Electroporation resulted in delivery of on average ∼60 labeled 4.5S RNA molecules per cell, which were subsequently diluted 4–16 times by cell division before data acquisition. Thus, the labeled 4.5S RNA molecules (5–15 molecules/cell) represent only a small fraction of the endogenous 4.5S RNA [∼1,000 molecules/cell (23)] and is therefore expected not to alter the overall SRP targeting pathway kinetics in the cells.

Small cell colonies were imaged under stroboscopic 1.5-ms laser exposures at 20-ms camera exposures. Fluorescence time-lapse movies were processed via a semiautomated analysis pipeline (19, 24), including cell segmentation, fluorescent dot detection, and diffusion trajectory building. The use of the LD655 dye [a Cy5 analog linked to a triplet state quencher (25)], in combination with an oxygen scavenging system (26), led to a fivefold increase in the photostability of the label compared to the common sulfo-Cy5 dye used in our previous tRNA tracking experiments (19). As a result, the experiments yielded ∼2 times more trajectory steps per cell and trajectories 1.5 times longer on average (*SI Appendix*, Fig. S5 and Table S1).

**Fig. 1.**
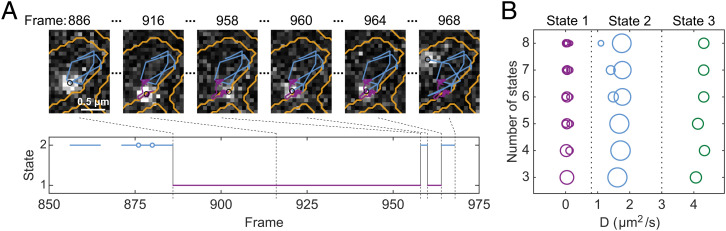
Tracking of single LD655-labeled 4.5S RNA in live *E. coli* cells. (*A*) Time lapse of 4.5S RNA tracking at 20 ms per frame. The diffusion trajectory is color-coded with respect to HMM estimated diffusion state (coarse-grained three states model). Cell outlines are shown in yellow and current position as a black circle. The lower part shows HMM estimated transitions between diffusion states. Blue circles represent missing positions in the trajectory building. (*B*) HMM fitting of 4.5S RNA diffusion trajectories to models of different sizes. The area of the circles is proportional to the occupancy of that particular state. Thresholds of 0.8 µm^2^/s and 3 µm^2^/s used for coarse graining are shown with vertical dashed lines.

The labeled 4.5S RNA displays apparent transitions between fast and slow diffusion regimes (Fig. 1 and Movie S1), suggesting dynamic binding to slow-diffusing ribosomes inside the cells. For analysis of single-particle trajectories, we applied a hidden Markov model (HMM)-based approach (24), which has previously been used successfully for analysis of diffusing molecules in vivo, transitioning between multiple binding states (19–22, 27). This HMM algorithm fits an ensemble of trajectories to a global model of discrete (hidden) states with different diffusion constants and stochastic (Markovian) transitions between these states. The algorithm further accounts for motion blur and localization uncertainties of each position and can handle missing positions. The algorithm searches for global maximum likelihood estimates of the fitted parameters for each considered model size (i.e., state occupancies, diffusion constants, and transition probabilities between these states).

Based on prior knowledge of the SRP pathway (3, 4), we expected to distinguish at least three separate diffusion states of the labeled RNA: free RNA, RNA in complex with Ffh (i.e., free SRP), and SRP bound to translating ribosomes. However, since the exact number of diffusion states fully describing the system is not known, and since there is, to our knowledge, no perfect statistical criterion to find the optimal number of diffusion states (see *SI Appendix, Supplementary Note 2*), we applied state models of size 3–8 in the HMM fitting procedure. We noticed that with increasing model size, the detected diffusion states are not uniformly distributed within the detection range but cluster in three distinct groups with diffusion coefficients 0.003–0.15 µm^2^/s, 1.1–1.8 µm^2^/s, and 4.1–4.3 µm^2^/s, respectively (Fig. 1*B*). Based on this clustering, we coarse grained models of eight states, down to three diffusion states, resulting in relative occupancy of the labeled 4.5S RNA in these states (i.e., the steady-state fraction) of ∼29% (state 1, 0.05 µm^2^/s), 56% (state 2, 1.7 µm^2^/s), and 16% (state 3, 4.3 µm^2^/s), respectively. It should be noted that the results and conclusions from this study do not depend on which initial model size (>3) was used for coarse graining (*SI Appendix*, Fig. S6 and Dataset S1). Coarse graining with the same threshold values was performed for all other experiments described below (*SI Appendix*, Fig. S7).

### Labeled 4.5S RNA Take Part in Ribosome Targeting.

In order to assign the different diffusion states to biologically relevant binding states of the 4.5S RNA, we first consider the diffusion coefficients of the individual states (Fig. 1*B*). Based on semiempirical models for macromolecular diffusion in the *E. coli* cytoplasm (28, 29), SRP (87 kDa) and free 4.5S RNA (37 kDa) would diffuse at around 1.2–1.8 µm^2^/s and 3.3–3.8 µm^2^/s, respectively. These numbers fall close to the fitted diffusion coefficients of state 2 (1.7 µm^2^/s) and state 3 (4.3 µm^2^/s). Hence, we tentatively assigned states 2 and 3 to free SRP and 4.5S RNA, respectively. The fitted diffusion rate of state 1 is 0.05 µm^2^/s. Considering that translating ribosomes have been reported to diffuse at 0.03–0.1 µm^2^/s (27, 30, 31), we tentatively assigned state 1 as ribosome-bound SRP.

**Fig. 2.**
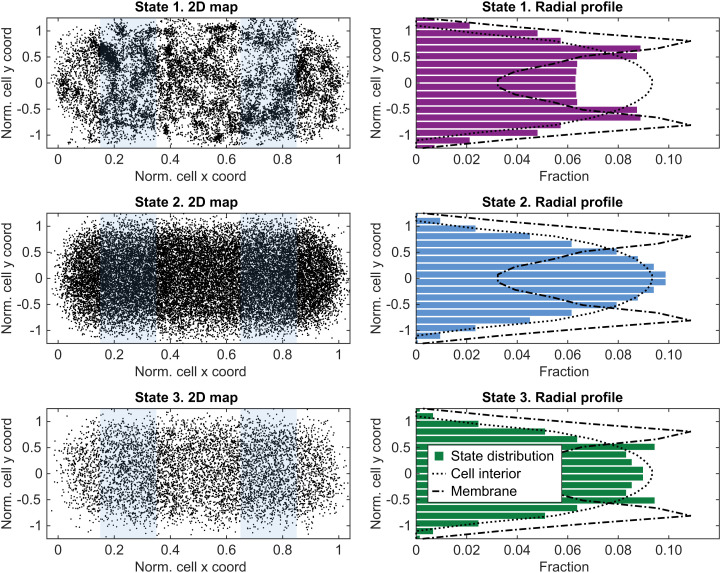
Spatial distribution of labeled 4.5S RNA in diffusion state 1 (ribosome-bound SRP), state 2 (free SRP), and state 3 (free 4.5S RNA). In the *left* panels, dot locations are plotted on normalized cell coordinates, and regions used for projection to the short cell axis are highlighted in blue. The *right* panels show the distribution of dot coordinates projected on the short cell radial axis. Dotted lines correspond to uniform distributions in the cytosol (theoretically predicted from the cell geometry), and dash-dotted lines correspond to membrane distribution (obtained by tracking the inner membrane protein LacY, *SI Appendix*, Fig. S8). Data in the *right* panel are mirrored across the long cell axis for better visibility. Nonmirrored data are available in *SI Appendix*, Fig. S9.

To investigate the spatial distribution of the respective diffusion states, we first benchmarked the theoretical profile for molecules distributed homogenously within a cell geometry (“cell interior” profile) (Fig. 2). As a benchmark for “membrane” spatial distribution, we performed single-particle tracking of labeled membrane protein LacY (Fig. 2 and *SI Appendix*, Fig. S8).

The spatial distributions of states 2 and 3 of labeled 4.5S RNA appear to be similar, with roughly homogenous distribution throughout the cell (Fig. 2 and *SI Appendix*, Table S2). State 1, on the other hand, seems to be distributed both in the cell interior and by the membrane (Fig. 2). Considering the radial distribution of state 1 as a superposition of “membrane” and “cell interior” components, we estimated that state 1 of 4.5S RNA consists of 44 ± 6% membrane-bound molecules (*SI Appendix, Supplementary Note 3* and Table S2). The same analysis applied to states 2 and 3 resulted in 0 ± 4% and 1 ± 3% membrane-bound molecules, respectively. Since the diffusion of the SRP–ribosome complex might be expected to change upon membrane binding, we were initially hoping to be able to separate these two biologically distinct states in the HMM analysis. Indeed, the results of multiple-state HMM fitting do suggest that state 1 actually represents a somewhat wider distribution of diffusions (Dataset S1), possibly reflecting two or more biologically distinct states. However, based on our extensive evaluation of simulated microscopy data (described below), we found that with our current 2D tracking approach and an HMM analysis pipeline that considers only diffusion trajectory step lengths and not positions relative to the cell geometry, we cannot reliably distinguish molecules with similar diffusion coefficients close to or bound to the cell membrane. Hence, in order not to introduce a known uncertainty into our analysis, we relied on the HMM results for the wider, coarse-grained “state 1”, which were then further supplemented with spatial distribution data, as discussed below.

**Fig. 3.**
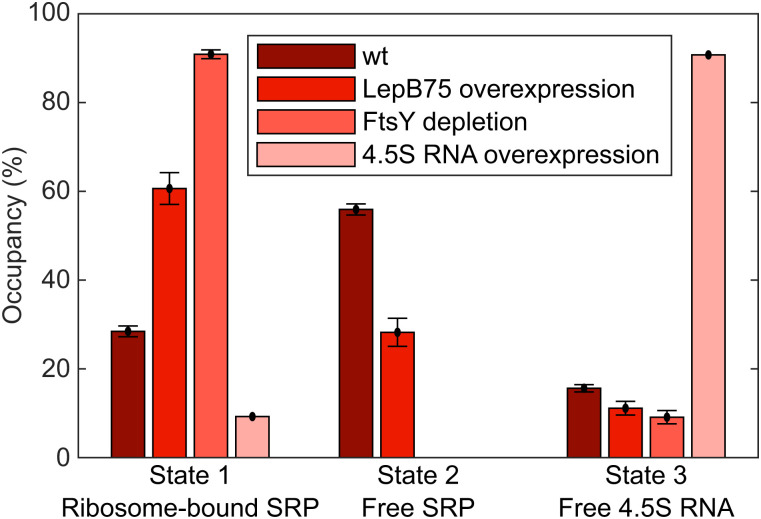
HMM estimated steady-state occupancy in the different diffusion states for wt cells (DH5α), cells overexpressing SRP-targeted LepB protein (first 75 aa) or nonlabeled 4.5S RNA, and cells depleted of the SRP receptor FtsY. Absence of bars in state 2 for FtsY depletion and 4.5S RNA overexpression is due to zero occupancy in these states.

To test whether the labeled 4.5S RNA actually participates in ribosome targeting, and to validate our state assignment, three critical control experiments were performed. First, we tracked the labeled 4.5S RNA in cells overexpressing 4.5S RNA, with the hypothesis that the additional nonlabeled 4.5S RNA would compete with the labeled 4.5S RNA to form SRP. While occupancy of state 3 is 16% in unmodified cells, it increased to ∼90% in cells overexpressing nonlabeled 4.5S RNA (Fig. 3). Hence, we concluded that state 3 represents free 4.5S RNA and that labeled 4.5S RNA experiences no or very little unspecific bindings.

In the second control experiment we instead wanted to trap the labeled 4.5S RNA in a functional state. To achieve this, we overexpressed a known SRP target peptide, the first 75 amino acids of leader peptidase, LepB75 (16). In this case, the 4.5S RNA should be involved in SRP binding to translating ribosomes to a higher extent. In the experiment, we observed a significant increase in occupancy of the hypothetical ribosome-associated state 1 (∼60%), compared to the background strain (∼30%) (Fig. 3). Hence, the experiment performed in LepB75-overexpressing cells confirmed the assignment of state 1 as a ribosome-bound state and, together with the fact that state 1 has a significant degree of membrane localization (Fig. 2), suggested that the labeled 4.5S RNA do take part in ribosome binding and translocon targeting in vivo.

Finally, to disrupt the potential translocon targeting of SRP ribosomes, we performed 4.5S RNA tracking in FtsY-depleted cells. The SRP receptor, FtsY, is needed for efficient delivery of SRP ribosomes to translocons (3, 4), and hence its depletion should prevent, or at least considerably prolong, the targeting time, and the labeled 4.5S RNA should be trapped longer in the ribosome-bound state. To test this, we electroporated labeled 4.5S RNA into an *E. coli* strain in which FtsY expression was under control of an arabinose-inducible promoter. In the absence of arabinose, and hence without further production of any FtsY, the cells became elongated and developed visible dark spots in the poles and by the membrane (*SI Appendix*, Fig. S12), possibly representing aggregates of nontargeted and hence incorrectly folded membrane proteins. From the single-molecule tracking results in these cells, we found almost 90% of the labeled 4.5S RNA in the proposed ribosome-bound state 1 (Fig. 3), in line with our expectation. This result provides additional support for correct assignment of state 1 as the SRP–ribosome complex. Furthermore, from the spatial distribution of 4.5S RNA in the FtsY-depleted cells, we found that the trajectory segments assigned to the slowly diffusing state 1 were evenly distributed inside the cell (*SI Appendix*, Fig. S13) instead of displaying a roughly 1:1 mixture of membrane and cytosolic distribution as in the wild-type strain, as described above. Thus, our 4.5S RNA tracking results from FtsY-depleted cells confirm a crucial role of FtsY as a membrane anchor for the SRP–ribosome complex.

### Kinetics of SRP-Mediated Ribosome Targeting.

To investigate the dynamics of the SRP cycle, we examined the transition frequency matrix estimated from the HMM analysis of 4.5S RNA tracking in wild-type cells (Dataset S1). The elements of the transition frequency matrix represent the calculated probabilities of transitions between the different states during each frame time (i.e., 20 ms). Hence, the HMM analysis provides us with an estimate of the underlying reaction scheme of the tracked molecules. By calculating state dwell times from transition probabilities, rather than measuring dwell times from individual complete binding-release events, we are not limited by photobleaching of the dyes and hence are not biased toward short events. It should here also be noted that we found that the HMM fitted transitions were self-consistent in terms of steady state. That is, the flux (state occupancy multiplied by the transition probability) of molecules into a particular state is, within the error estimate, the same as the flux out of that state (Dataset S1), even though this is not a constraint implemented in the HMM fitting algorithm.

We observed that the free 4.5S RNA only rarely converts to other states (the transition frequency is more than one order of magnitude lower than between other states; Dataset S1). Hence, 4.5S RNA seems to bind stably to Ffh, and little or no exchange of the RNA component of the SRP complex occurs. Based on transition probabilities between the two remaining states we found that SRP spends on average 1.9 s freely diffusing and about 1 s bound to a ribosome. These numbers remained the same, within 20%, independent of the HMM model size used for coarse-graining (*SI Appendix*, Fig. S6). In the FtsY-depleted strain, the dwell time in the ribosome-bound state was prolonged drastically, from 1 s to ∼19 s (Dataset S1), in line with our expectation of disrupted translocon targeting in this strain.

**Fig. 4.**
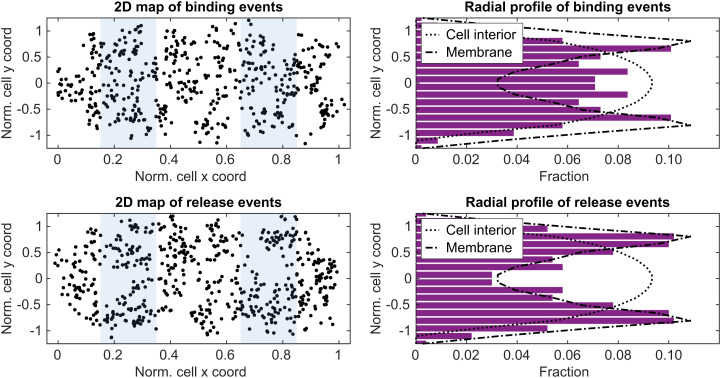
Spatial distribution of SRP–ribosome binding and release events detected as transitions to/from diffusion state 1 of labeled 4.5S RNA. In the *left* panels, locations of ribosome binding (*Upper*) and release (*Lower*) events are plotted on normalized cell coordinates. Regions used for projection to the short cell axis are highlighted in blue. The *right* panels show the distribution of binding and release events projected on the short cell radial axis. Dotted lines correspond to uniform distributions in the cytosol (theoretically predicted from the cell geometry), and dash-dotted lines correspond to membrane distribution (obtained by tracking the inner membrane protein LacY, *SI Appendix*, Fig. S8). Data in the *right* panels are mirrored across the long cell axis for better visibility. Nonmirrored data are available in *SI Appendix*, Fig. S14. *N*_arrivals_ = 184, *N*_departures_ = 201.

Since the HMM analysis does not consider positions of diffusion trajectories relative to the cell geometry, we next sought to capture spatial features of SRP-mediated targeting of ribosomes by mapping those HMM-assigned state transition events back onto the cells. Hence, to investigate the spatial location of transition events between the ribosome-bound and free SRP states, we plotted coordinates of SRP binding and release from the ribosome in internal cell coordinates and constructed the corresponding projections to the short cell axis (Fig. 4). From this radial distribution it is clear that bindings and release events are distributed differently in space. SRPs dissociate from ribosomes virtually exclusively by the membrane, whereas ribosome bindings occur both in the cell interior and directly by the membrane. Fitting the binding spatial distribution to a combination of “membrane” and “cell interior” profiles, we found that in 25 ± 9% of the cases, SRP binds to membrane-localized ribosomes, while in 75 ± 9% it binds to ribosomes in the cytosol (*SI Appendix, Supplementary Note 3* and Table S2). In both cases, however, SRP dissociates from the ribosome by the membrane, as seen from the spatial distribution of release events (estimated membrane fraction is 86 ± 7%, *SI Appendix, Supplementary Note 3* and Table S2). Finally, by separating ribosome binding events occurring in the cytosol and by the membrane, we also found that once the complex has reached the membrane, it stays there, irrespective of initial binding mode (cytosol or membrane proximate), until SRP dissociates from the ribosome (or as long as the photobleaching-limited window allows observation) (*SI Appendix*, Fig. S15).

**Fig. 5.**
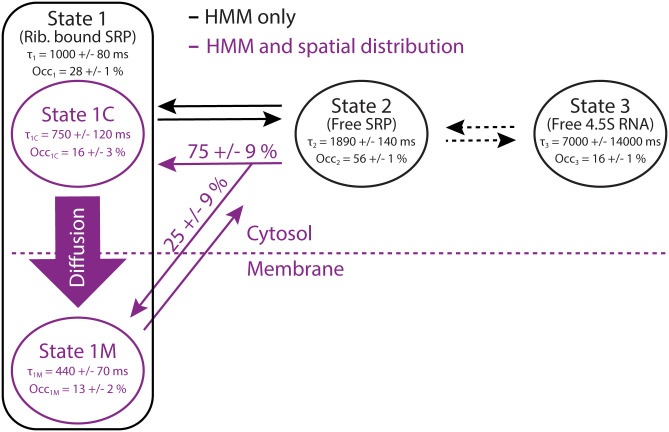
Reaction–diffusion model of 4.5S RNA interactions in *E. coli* derived from experimental single-molecule tracking data. State 3 (free 4.5S RNA) and state 2 (free SRP) are diffusing in the cytosol compartment, while state 1 (ribosome-bound SRP) is located in both cytosol (C) and membrane (M) compartments. “Occ” is state occupancy, and “τ” is state dwell time. Data shown in black were obtained from HMM analysis, while data shown in purple were calculated based on the HMM analysis results in combination with the spatial distribution profiles of total occupancy and binding/release events (*SI Appendix*, Table S3).

Based on our results, now including both temporal and spatial information, we constructed a putative kinetic model for the SRP cycle, which allows two types of SRP–ribosome binding modes—anywhere in the cytosol or directly by the membrane, with SRP release from ribosomes only by the membrane (Fig. 5). The model further assumes the same mean dwell time for the SRP–ribosome complex by the membrane for both binding modes, although we cannot exclude that these times are different. However, the suggested model is the simplest model we can find that would give rise to the experimental data at hand. Based on the HMM estimated diffusion state occupancies and dwell times (*SI Appendix*, Table S3 or Fig. 5), in combination with information from the analysis of spatial distributions (Figs. 2 and 4), we estimated that SRP spends 440 ± 70 ms bound by the membrane and that the membrane search time by the 3D diffusing ribosome–SRP complex is 750 ± 120 ms (Fig. 5).

### Simulated Microscopy Refines and Validates Experimental Results.

To quantitatively validate the reaction–diffusion model of the SRP cycle derived from our tracking experiments (Fig. 5) and to investigate the precision of our analysis, we then turned to simulated microscopy (the workflow is explained in *SI Appendix*, Fig. S16). In short, we generated particle trajectories by simulating a ground truth reaction–diffusion model inside an *E. coli* cell geometry using the MesoRD software (32). The obtained diffusion trajectories were then used to simulate microscopy data in the SMeagol software (33) with an experimentally derived point spread function and sample background noise. Vertical (*z* coordinate) cell-to-cell variation of the cell middle plane, and *x*–*y* coordinate cell segmentation errors, were also simulated to get the apparent spatial distribution of membrane molecules similar to the experimentally acquired LacY spatial distribution (*SI Appendix*, Fig. S17). The in silico generated datasets were finally analyzed through the same image analysis and HMM pipeline as used for in vivo experimental data.

The initial reaction–diffusion model in the simulations was based on the model constructed from our in vivo tracking experiments (Fig. 5), with diffusion coefficients and reaction rates in the model then being iteratively adjusted in order to achieve best agreement between in silico and in vivo experimental results (i.e., HMM-estimated diffusion coefficients, occupancies, and dwell times, as well as the spatial distribution of binding events). The ground truth and HMM estimated parameters of the simulated model, along with in vivo results, are shown in *SI Appendix*, Table S3.

From our simulations, we first concluded that the analysis pipeline, including dot detection, trajectory building, and finally HMM fitting, is capable of finding the underlying kinetic model within reasonable precision. However, we found that the diffusion coefficients were underestimated (30% for states 1 and 2, 90% for state 3), probably because of confinement effects (34). We also found that the dwell time in diffusion state 1 was systematically overestimated by up to ∼36% (*SI Appendix*, Table S3). We speculate that this discrepancy stems from difficulties in the HMM analysis to distinguish some of the state transitions due to the confined cell geometry, particularly near the membrane (i.e., the missing *z*-coordinate tracking have much higher influence during 2D diffusion in the membrane than during 3D diffusion in the cytosol). Indeed, a simpler model without membrane binding yielded a lower bias of dwell times (∼26%, *SI Appendix*, Table S4). Taking these limitations into account, however, we can suggest a detailed reaction–diffusion model that, according to our simulations, would give rise to the experimentally derived parameters. That is, assuming the dual-pathway model for SRP-mediated targeting of ribosomes to translocons, in accordance with the experimentally determined spatial distribution of SRP–ribosome binding events (Fig. 4 and *SI Appendix*, Fig. S15), we found that it takes on average 1.5 s for SRP to find a target ribosome and furthermore that the SRP–ribosome complex spends on average ∼350 ms during 3D random diffusion search for the membrane and an additional ∼500 ms by the membrane before SRP dissociates. Approximately 75% of the SRP–ribosome binding events occur anywhere in the cytosol, whereas the remaining 25% of binding events instead occur directly by the membrane. The mean dwell time for SRP in the ribosome-bound state (considering both binding pathways) is ∼750 ms (*SI Appendix*, Table S3).

Finally, by analyzing datasets of different sizes, we found that the HMM analysis converges when datasets consist of about 40,000 trajectory steps or more (*SI Appendix*, Fig. S18), which was fulfilled in our experiments.

## Discussion

We tracked dye-labeled 4.5S RNA in live *E. coli* cells and were able to follow SRP-mediated targeting of ribosomes to translocons in space and time. Thanks to the improved photostability of the triplet-state-quencher fused dye, we could track single SRP molecules up to more than 100 frames and observe transitions between different functional states. Hence, in comparison to previous SRP single-molecule tracking approaches, relying on fluorescent protein fusion to Ffh (6), our method allows high-precision estimates of the time SRP molecules spend in different diffusion states.

A limitation of our HMM analysis approach is that biologically distinct binding states that have similar diffusion coefficients (e.g., the SRP–ribosome–mRNA complex docked or not docked to a translocon) cannot always be reliably separated and that the spatial location of molecules is not considered during HMM fitting. In order to overcome this problem with respect to the SRP–ribosome complex, we went back to the raw microscopy data to map states and state transitions found in the HMM analysis onto the actual geometry of the cell. This way, a picture emerged showing that state transitions were not uniformly distributed within the cell but rather that there was a clear pattern of physical movement during the targeting process (Fig. 4). This combined analysis resulted in a spatiotemporal model of the SRP pathway (Fig. 5), which was further refined via simulated microscopy.

We found that free 4.5S RNA forms a stable SRP complex with Ffh once it is bound and that free SRP spends on average 1.5 s freely diffusing throughout the whole cell before finding a target ribosome. From in vitro experiments in reconstituted systems, it has been suggested that SRP binds transiently and indiscriminately to practically all ribosomes (35, 36). Since the actual target ribosomes constitute maximally only a few percent of the total elongating ribosomes (37), and since the absolute majority of the SRP–ribosome binding events found in our analysis end by the membrane (Fig. 4), we conclude that our tracked SRP–ribosome binding events represent actual targeting events of ribosome–nascent peptide complexes and that SRP unproductive sampling of elongating ribosomes must be faster than the time resolution of our experiments (i.e., less than ∼40 ms). This is shorter than previous estimates of SRP dwell times on nontarget ribosomes, 70–100 ms, estimated in ensemble biochemical experiments (35), but in line with the conclusions from in vitro single-molecule Förster resonance energy transfer experiments, where no such sampling events were detected at a time resolution of 100 ms (38). Considering the limited number of SRPs inside the cell, which have to quickly sample a much higher number of ribosomes [SRP/ribosome ratio estimated as 1/100 (39)], such short sampling times also make perfect sense. Assuming that 10% of all elongating ribosomes synthesize SRP-targeted IMPs (40), and that the window for SRP binding is about 10% of the total translation time [20 aa of an average 200-aa protein (1)], we estimate that ∼1% of the ribosomes should at any given moment represent actual targets for SRP. If on average 99 ribosomes are to be probed to find one correct target ribosome within our estimated search time, 1.5 s, this then suggests that the sampling time per incorrect ribosome cannot exceed 15 ms.

We differentiated two pathways for translocon targeting of ribosomes by SRP. In the first path (∼75% of binding events), SRP binds to a ribosome in the cell cytosol and deliver it to the membrane by 3D diffusion. In the second path (∼25% of binding events) SRP binds to a ribosome already located by the membrane. Based on our single-molecule tracking data, refined by simulations, we found that the 3D diffusion process takes on average 350 ms and that the SRP–ribosome complex spends on average ∼500 ms by the membrane (*SI Appendix*, Table S3). Considering the complexity of the mechanism, it is difficult to compare these compounded average times to kinetic estimates of individual steps of the targeting process acquired in reconstituted systems. However, our observations definitely suggest a much more rapid transfer process in vivo than, for example, the 70- to 140-s targeting time deduced from in vitro fluorescence and Förster resonance energy transfer experiments (41). Hence, although such in vitro experiments are crucial to connect macromolecular structure with function, the absolute times measured were in this case probably unphysiological (as already noted by the authors of that study), which also underscores the importance of the present kinetic measurements directly inside living cells.

Unless membrane-proximate targeting is, on average, considerably faster than the time resolution of our experiments, ∼40 ms, so that our detected spatial distribution of ribosome binding events (Fig. 4) is biased, our observations suggest that a majority (∼75%) of targeting events start in the cytosol. Considering findings from proximity-specific ribosome profiling in eukaryotic cells, where it was concluded that the mRNA tethered state is crucial for efficient cotranslational targeting (42), this result is rather surprising and has some interesting consequences. Since a translocon-targeted ribosome will pull the mRNA along with it, it is tempting to speculate that ribosome binding events starting in the cytosol represent targeting of pioneer ribosomes on open reading frames (ORFs). If so, the rather high fraction of these events might suggest that SRP is not needed in general for targeting of trailing ribosomes. However, our experimentally derived kinetic measurements do not support this hypothesis. That is, based on known IMP concentration, SRP concentration, and cell generation time, we can estimate that the required SRP cycling time in *E. coli* should be about 2–3 s at this growth rate, if all IMPs are targeted by SRP (see *SI Appendix*, Table S5 for calculation). This number is in line with the average SRP cycling time estimated from our single-molecule experiments, ∼2.2 s, and therefore shows that the frequency of SRP-mediated ribosome targeting observed in our experiments is consistent with the expected frequency if all IMP-translating ribosomes would need SRP. The fact that our observed ribosome targeting frequency corresponds well with the required targeting frequency, in addition, suggests that we do not miss a considerable amount of targeting events due to the time resolution of our experiment. Hence, we propose that the observed 75% of binding events in the cytosol instead represent targeting of both pioneer and trailing ribosomes and thus that membrane tethering of mRNA is neither important nor prevalent for SRP-mediated ribosome targeting in *E. coli*, in contrast to the findings in eukaryotic cells (42). Ribosome profiling results from *E. coli*, suggesting comparably low ribosome density on IMP-encoding ORFs (43), provide a clue as to how this could be: With sparsely distributed ribosomes, the 5′ end of the ORF will have time to diffuse away from the membrane in between most of the targeting events. Hence, in a small bacterial cell, such as *E. coli*, the relatively short 3D search time from anywhere inside the cell to the membrane, 350 ms, in practice makes each SRP-mediated ribosome targeting an independent event.

If the larger fraction of binding events, 75%, represent independent targeting of ribosomes from the cytosol, the remaining 25% occurring directly by the membrane could then represent a lower fraction of trailing ribosomes translating mRNAs already tethered to the membrane; rebinding of SRP to ribosomes during insertion of internal transmembrane domains [in line with the findings in (1)]; translocon targeting of ribosomes and mRNA delivered to the membrane independent of SRP (7); or a mixture of two or three of these scenarios. Additional experiments (e.g., tracking of individual mRNAs) will be needed to settle this definitely.

**Fig. 6.**
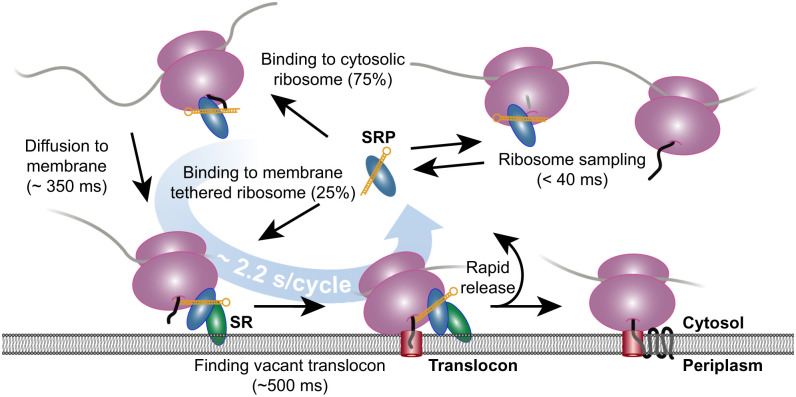
Proposed kinetic model for SRP-mediated targeting of nascent peptide–ribosome complexes to membrane-bound translocons in *E. coli*, derived from experimental single-molecule tracking data refined by microscopy simulations.

Our data further show that SRP lingers on the membrane-associated ribosome for a significant fraction of its bound time (500 ms vs. 350 ms during 3D diffusion). In combination with our results showing that the SRP receptor, FtsY, is crucial for the SRP–ribosome–membrane association, we suggest that a substantial fraction of this membrane-bound time is spent searching or waiting for a vacant translocon and that FtsY hence serves as an anchor for membrane association of the SRP–ribosome complex. With an estimated FtsY copy number higher than that of translocons (23, 40) and translocons being highly occupied by both posttranslational and cotranslational polypeptide insertion, this strategy would significantly speed up the translocon search process, practically reducing the 3D search problem to one in 2D. On the other hand, if the targeting process involves only a pure 3D search, and our measured 500 ms by the membrane instead represents some structural rearrangement of the complex already attached to the translocon, or, for example, a rather slow GTP hydrolysis step, we see no added benefit of having a highly abundant separate SRP receptor. Hence, based on our SRP tracking results, in the presence and absence of FtsY, and on estimated copy numbers of the individual components of the pathway, we conclude that the most likely scenario would be that the 3D diffusing SRP ribosome first attaches to the membrane with the help of the SRP receptor FtsY. By the membrane, it takes on average ∼500 ms to finish the targeting process, including 2D diffusion to find or wait for a vacant translocon, where structural rearrangements and finally GTP hydrolysis on SRP and FtsY trigger the release of SRP (Fig. 6). It remains to be elucidated, however, whether FtsY associates to the SRP–ribosome complex already during the 3D search process in the cytosol (41) or whether it serves as an abundant docking partner attached to the membrane (44).

Finally, from SRP-specific ribosome profiling experiments (1), an average SRP footprint length of 11 codons can be deduced (*SI Appendix, Supplementary Note 4*). In the study, there was also no evidence of translation slowdown on SRP target ORFs, as has been suggested previously (45). From this type of ensemble experiment, however, it is not possible to tell whether the footprint represents a distribution of many short binding events or whether every single binding event persists for on average 11 codons. From our single-molecule tracking-derived dwell time of SRP on ribosomes, ∼750 ms, we estimate that the nascent polypeptide is elongated on average 12–13 aa during each translocon targeting event, assuming an average elongation rate of 16–17 aa/s at a growth rate of 1.3–2.6 dbl/h (46, 47). This number is hence in perfect line with the average ribosome profiling based SRP footprint length of 11 codons, thus showing that the aa-length distribution from the ribosome profiling data mainly represents the distribution of single SRP binding events and not a sum of consecutive binding events (*SI Appendix*, Fig. S19). This result also provides a clue as to why the *E. coli* SRP does not possess peptide elongation stalling capability (1, 5), as is the case for SRP-mediated endoplasmic reticulum targeting in eukaryotes (48); an extension of the polypeptide with merely 12–13 aa on average before the ribosome docks to the translocon is probably not enough to cause any significant polypeptide solubility issue in most cases. Hence, the stalling capacity, or even translation slowdown, is not needed in a small *E. coli* cell. However, upon overloading of the translocons (e.g., during membrane protein overexpression), the search time for a vacant translocon is expected to be higher and hence might lead to protein misfolding problems.

In conclusion, by combining single-particle tracking, numerical modeling, and simulated single-particle tracking, we have presented a quantitative reaction–diffusion model for SRP-mediated cotranslational targeting of ribosome–nascent peptide complexes to membrane-bound translocons in living *E. coli* cells. Based on our results, we suggest that SRP–ribosome–nascent peptide complexes find their target through a combination of 3D and 2D search, where only approximately a quarter of the targeting events occur on ribosomes already tethered to the membrane. Finally, the validation of our results through simulated microscopy underscores the potential of single-particle tracking in studies of complex intermolecular networks directly inside living cells.

## Materials and Methods

### 4.5S RNA Purification, Labeling, and Gel Mobility Shift Assay.

Expression of 4.5S RNA was performed as described previously (49). *E. coli* DH5α cells carrying the pSN1 plasmid were grown for 8 h in 1 L of Luria–Bertani medium supplemented with 100 µg/mL ampicillin and 1 mM isopropyl β-D-1-thiogalactopyranoside (IPTG). Cells were harvested and resuspended in 20 mM potassium acetate (pH 4.7) containing 1 mM ethylenediaminetetraacetic acid (EDTA), and RNA was extracted with a phenol/chloroform/isoamyl alcohol solution (125:24:1), pH 4.5 (Invitrogen) and precipitated with sodium acetate in isopropanol. Purification of 4.5S RNA was performed by reversed-phase high-performance liquid chromatography with a LiChrospher WP 300 RP-18 (5 µm, 250 × 4 mm) column, where the bound material was eluted at 22 °C at a flow rate of 0.5 mL/min with a linear gradient of methanol (from 15% to 72% in 60 min) in buffer containing 20 mM ammonium acetate, 10 mM MgCl_2_, and 400 mM NaCl, pH 5.0. Collected fractions were concentrated with Amicon Ultra 0.5 mL centrifugal filters (Merck Millipore) with a 3-kDa molecular weight cutoff, precipitated with ammonium acetate in ethanol, redissolved in water, and stored at −20 °C.

4.5S RNA was fluorescently labeled at the 3′ end with LD655 dye (Lumidyne Technologies) in two steps. First, 6.4 nmol of 4.5S RNA was 3′ ribose oxidized by incubation for 35 min on ice in the dark in a solution containing 100 mM sodium acetate (pH 5.0) and freshly dissolved 5 mM potassium periodate. The reaction was quenched by addition of ethylene glycol to a final concentration of 10 mM, followed by incubation for 5 min on ice. Oxidized 4.5S RNA was precipitated twice with ammonium acetate in ethanol and dissolved in labeling buffer (100 mM potassium acetate, 200 mM KCl, pH 5.0). Second, 30 µL of 107 µM oxidized 4.5S RNA was mixed with 1.5 µL of 10 mM LD655 monoreactive hydrazide dye dissolved in anhydrous dimethyl sulfoxide and the reaction mixture was incubated at 22 °C for 5 h, with occasional vortexing. Unreacted dye was extracted with a phenol/chloroform/isoamyl alcohol solution (125:24:1), pH 4.5 (Invitrogen), followed by precipitation of 4.5S RNA from aqueous phase with ammonium acetate in ethanol. The pellet was washed with 70% ethanol, dried, redissolved in water, and stored at −80 °C. The labeling yield was ∼50%.

Fluorescently labeled 4.5S RNA was isolated by reversed-phase high-performance liquid chromatography according to the procedure for 4.5S RNA isolation as described above, dissolved in 5 mM ammonium acetate (pH 5.5), and stored at −80 °C. The purity of fluorescently labeled 4.5S RNA and capability to bind Ffh was analyzed by native polyacrylamide gel electrophoresis.

The gel mobility shift assay was performed with a nondenaturing polyacrylamide gel with labeled 4.5S RNA, as well as nonlabeled periodate oxidized 4.5S RNA, with or without Ffh. SRP was reconstituted in 10 µL of binding buffer (20 mM HEPES–KOH, 10.6 mM magnesium acetate, 100 mM ammonium chloride, 0.5 mM EDTA, pH 7.1) with 1 µM 4.5S RNA and 3 µM Ffh. After 20 min of incubation at 25 °C, 3 µL of 60% glycerol was added and 5 µL of the sample was loaded on a 1 × 83 × 83 mm 7% polyacrylamide gel (19:1 acrylamide:bisacrylamide). The electrophoresis buffer (pH 6.5) contained 50 mM Tris acetate, 75 mM ammonium acetate, 10 mM magnesium acetate, and 1 mM EDTA. Gel electrophoresis was run at room temperature in a Bio-Rad Mini-PROTEAN Tetra Cell at 100 V for 70 min and was then stained with SYBR Gold and imaged in a Bio-Rad ChemiDoc imaging system. The Ffh protein was expressed from a pDMF6 plasmid (pET3c vector) in *E. coli* BL21 (DE3) pLysE strain as described elsewhere (49).

### Cell Strains Used in Microscopy Experiments.

All experiments except FtsY depletion were performed with *E. coli* DH5α strain. Overexpression of 4.5S RNA was performed in cells containing pSN1 plasmid (49). Overexpression of the LepB75 fragment was performed in *E. coli* DH5α strain carrying plasmids pET28a–LepB75 and pCS6 (Addgene #55752). The pET28a–LepB75 plasmid was constructed by insertion of the N-terminal 75 aa of the LepB protein in a pET28a vector (Novagen). For imaging of cell membrane, a LacY–HaloTag fusion with Gly-Ser-Gly linker was inserted into a modified pQE-30 (Qiagen) vector via a NEBuilder HiFi DNA assembly protocol, resulting in the pQE30Mod–lacY–Halotag plasmid (full sequence provided in the SciLifeLab Data Repository, see section *Data, Materials, and Software Availability*). Imaging was performed in *E. coli* DH5α carrying the pQE30Mod–lacY–Halotag plasmid.

For electroporation of 4.5S RNA, DH5α cells were made competent as described in (19, 20), or ElectroMAX DH5α-E competent cells (Invitrogen) diluted in 10% glycerol (5× dilution) were used.

For the FtsY depletion experiment, the *E. coli* strain BW25113–Kan–AraCP–ftsY (Eitan Bibi laboratory), in which expression of the ftsY gene is regulated by an arabinose promoter, was grown on LA (Luria Agar) plates supplemented with 50 µg/mL kanamycin and 0.1% arabinose. A single colony was used to inoculate 100 mL of SOB (Super Optimal Broth) without Mg^2+^, containing 0.1% arabinose, and the culture was grown at 37 °C, 200 rpm. Cells were grown to an optical density at 600 nm (OD_600_) of ∼0.1 and pelleted by centrifugation at 3,000 × *g*. The pellet was washed in 10 mL of SOB without Mg^2+^, and cells were transferred into 100 mL of SOB without Mg^2+^ containing 0.2% glucose to suppress production of ftsY and grown for additional 1 h at 37 °C, 200 rpm. Cells were made competent and stored according to the protocol described elsewhere (19, 20).

### Cell Electroporation and Microscopy Sample Preparation.

#### Delivery of labeled 4.5S RNA.

Electroporation of 4.5S RNA was performed as described previously (19). In brief, competent cells (20 µL) were mixed with 1 pmol of dye-labeled 4.5S RNA and electroporated in a 1-mm electroporation cuvette (Thermo Scientific) with a MicroPulser Electroporator (Bio-Rad) at a voltage of 1.9 kV (1.8 kV for FtsY depletion strain). Cells were recovered in 0.5 mL of EZ RDM with 0.2% glucose (Teknova) at 37 °C for 30 min and washed three times with RDM to remove noninternalized dye-labeled 4.5 S RNA (i.e., centrifuging 2 min at 1,200 × *g*, followed by supernatant removal and dilution in fresh 0.5 mL of 37 °C RDM). Cells were diluted to OD_600_ = 0.03 in RDM and sparsely spread on a 2% agarose pad to achieve a sample with well-separated individual cells. The agarose pad was prepared with RDM and SeaPlaque GTG Agarose (Lonza) and supplemented with 1 µM dead cell stain SYTOX Blue (Invitrogen). To improve photostability of the LD655 dye, the agarose was also supplemented with an oxygen scavenging system consisting of 2.5 mM protocatechuic acid (Sigma, 100 mM stock stored frozen in water–NaOH, pH 8) and 0.05 U/mL protocatechuate 3,4-dioxygenase (OYC Americas). The agarose pad was surrounded with a gene frame (Thermo Fisher) and sealed between the microscope slide and the coverslip (#1.5H, Thorlabs). The sample was mounted on the microscope, where single cells were grown to colonies of 4–16 cells and imaged at 37 ± 2 °C.

For 4.5S RNA overexpression experiments, the agarose pad was additionally supplemented with 1 mM IPTG and 1 mM cyclic adenosine monophosphate, whereas for LepB75 overexpression experiments, the agarose pad was supplemented with 30 µM IPTG and 0.2% arabinose.

For comparative photostability testing, 4.5S RNA was labeled by sulfo-Cy5 hydrazide dye (GE Healthcare) and purified as described above. Sample preparation and microscopy were essentially the same as for LD655 sample, except that no oxygen scavenging components were added on the agarose pad when sulfo-Cy5 labeled 4.5S RNA was used.

#### Labeling of LacY–HaloTag.

DH5α cells carrying the pQE30Mod–lacY–Halotag plasmid were grown overnight in EZ RDM with 0.2% glucose (Teknova) supplemented with carbenicillin (100 µg/mL) in a 37 °C shaking incubator. The culture was diluted 50 times in 7 mL of fresh RDM with carbenicillin and grown to OD_600_ of ∼0.5–1. Cells were centrifuged (3 min, 2,800 × *g*) and resuspended in 150 µL of RDM. To ensure similarity in point spread functions, which are wavelength-dependent and may potentially affect the 2D profile of 3D distributed molecules, we used the JF646 HaloTag dye for LacY labeling, which is spectrally similar to the LD655 dye used for 4.5S RNA tracking. JF646 HaloTag dye, initially dissolved in dimethyl sulfoxide, was added to a final concentration of 0.05 µM, and cells were incubated at 25 °C. After 30 min, cells were washed three times with 1 mL of M9 glucose medium. Here and in later steps Eppendorf tubes were changed after each washing to avoid dye absorbed onto the plastic tubes. Cells were resuspended in 1 mL of RDM, transferred to a polystyrene culture tube, and incubated in a 37 °C shaking incubator for release of nonreacted dye. After 40 min, double washing in M9 glucose was repeated, and cells were resuspended in RDM and finally sparsely spread on an agarose pad (prepared with only RDM). No induction with IPTG was done since the leakage level of LacY–HaloTag was enough to get desired number of labeled molecules.

Each microscopy experiment was performed in two or three replications (one for LacY–HaloTag tracking), each comprising 100–300 cell colonies with internalized labeled 4.5S RNA. The results were found consistent in between repetitions and were combined for analysis.

### Optical Setup.

Widefield epifluorescence microscopy was performed on a Nikon Ti-E inverted microscope equipped with a CFI Apochromat TIRF 100XC Oil 1.49 NA objective (Nikon). Bright-field and fluorescence images were recorded with an EMCCD iXon 897 Ultra camera (Andor) connected through an additional magnifying 2.0× camera adapter (Diagnostic Instruments). An Infinity2-5 M camera (Lumenera) was used for phase contrast imaging. Tracking of LD655-labeled 4.5S RNA was carried out with a 639 nm Genesis MX 639–1000 STM laser (Coherent) with a power density of 5 kW/cm^2^ on the sample plane in stroboscopic illumination mode with 1.5-ms laser/20-ms camera exposure. The same setup but with 3-ms laser/20-ms illumination mode was used to record fluorescent images of JF646-labeled LacY–HaloTag. Cells stained with SYTOX Blue (dead cells) were detected with a 405-nm laser (Cobolt MLD) with a power density of 10 W/cm^2^ and exposure time of 21 ms. Data were automatically acquired with an in-house-made µManager plugin.

### Data Analysis.

Data were analyzed via a previously described MATLAB-based pipeline (19). Cell outlines were detected based on phase contrast images via a fast adaptive local thresholding algorithm (50). Segmentation masks were rescaled and overlaid with bright field and fluorescence images with landmarks and autocorrelation procedures and finally curated semiautomatically to keep only cells that formed small colonies of 4–16 cells and discard incorrectly segmented cells, dead cells (bright under 405-nm laser illumination due to SYTOX Blue staining), and cells that did not contain labeled 4.5S RNA.

Bright spots representing single fluorophores were detected in the fluorescence channel via the radial symmetry–based algorithm (51). A Gaussian spot model and maximum posteriori fit (24) were used to refine spot positions, estimate position uncertainty, and filter out erroneously detected spots. Trajectories of individual molecules were constructed by linking their positions on consecutive frames via the u-track algorithm (52), allowing gaps with single missing points. In each segmented cell, trajectories were built from the timepoint when there was only one spot remaining in current and following frames (or two spots for experiments performed with LacY–HaloTag).

The resulting ensemble of trajectories for each experimental condition was analyzed via an HMM algorithm (24), according to the procedure described in (19), except that the subsequent pruning of states was not done. The HMM fitting procedure resulted in a model of fixed size with discrete diffusion states, characterized by diffusion coefficient, occupancy, and transition probabilities between these states. The obtained models (three to eight states) were coarse-grained to three states based on the apparent clustering of states with respect to detected diffusion coefficients, using thresholds 0.8 µm^2^/s and 3 µm^2^/s. Coarse-grained diffusion constants and occupancies were calculated as weighted averages, and coarse-grained mean dwell times were estimated from the coarse-grained transition matrix. A detailed list of parameters for data analysis is available together with raw data in the data repository (https://doi.org/10.17044/scilifelab.20502126).

LacY–HaloTag tracking data were analyzed with a two-state model, resulting in state 1 representing LacY diffusion and state 2 representing tracking artifacts.

For spatial occupancy plots, positions were classified as belonging to a certain (coarse-grained) state if the HMM posterior probability was >95%, whereas more ambiguous positions were excluded. Positions of ribosome binding and release (arrival and departure events to/from state 1) in the 4.5S RNA tracking data were determined from the HMM-fitted Viterbi path. To increase precision, two positions of the particle following the arrival event and two positions of the particle preceding the departure event (as explained in *SI Appendix*, Fig. S20) were also included in the construction of scattered plots and cross-section profiles (Fig. 4 and *SI Appendix*, Fig. S14). Given that state 1 (ribosome-bound SRP) is long lived (∼1 s) compared to frame intervals (20 ms), and diffusion is slow (∼0.05 µm^2^/s), these neighboring positions have almost identical spatial locations as the true arrival/departure positions. To avoid inclusion of “false” transitions that may occur due to tracking artifacts or imprecise assignment of states, only transitions for which the particle had a trajectory with at least three trajectory segments preceding and following the position of the transition were included in the analysis. Histograms of spatial distribution radial profiles (number of bins *n_b_* = 18) were constructed only for positions located in the cylindrical part of the cell. Thus, positions with long cell axis coordinate *x* in cell pole regions 0 < *x < m* and (1 − *m*) < *x* < 1, and in the middle of the cell, possibly containing the cell division septum, (0.5 *− m*) < *x* < (0.5 + *m*), where margin *m* = 0.15, were excluded. Results do not depend significantly on the choice of *m* and *n_b_* (*SI Appendix, Supplementary Note 3*).

### Simulation of Single-Molecule Microscopy.

Simulation of reaction–diffusion kinetics and video microscopy was performed similarly to that described in (19).

#### Simulation of reaction–diffusion kinetics with MesoRD (32).

The simulated cell geometry consisted of two compartments, the cytosol and the membrane. The cytosol was constructed from one cylinder of radius 0.42 µm and length 3 µm, with two half spheres of radius 0.42 µm as caps at the ends of the cylinder. The membrane compartment was constructed as a 0.05-µm-thick layer around the cytosol compartment. In the case of a simple single compartment model, the cytosol cylinder and the half-sphere caps had a radius of 0.47 µm. Width and length of the cell segmentation masks used for simulations were equal to the mean width and length of the segmentation masks obtained from image analysis of in vivo data. Four different molecule species were defined: RNA (i.e., 4.5S RNA), SRP, RC, and RM (i.e., ribosome-bound SRP in cytosol and membrane compartments, respectively). Conversion rates and allowed species location are shown in *SI Appendix*, Fig. S21.

Ten simulations were initiated, each with 200 molecules in the RNA state. The system was equilibrated for 56 s (simulated time) before trajectories and reaction data were acquired during an additional 20 s. The cube size of the simulated 3D system was 0.01 µm (making the membrane layer five cubes thick), and the time step was 0.0057 s. Trajectories from the MesoRD simulations were randomly reordered, and Z and X–Y “noises” were added on a trajectory-to-trajectory basis to mimic cytosolic and membrane special distributions of molecules obtained from in vivo data (*SI Appendix*, Fig. S17). Cell-to-cell normally distributed (std 100 nm) Z variation of trajectory coordinates was introduced to mimic the experimental situation when cells on the agarose pad have slightly different Z positions relative to the sample plane and when different positions on the agarose pad were acquired with a slightly different Z offset. This Z variation may change the appearance of the molecules in the detector due to the finite depth of the microscope point spread function, which is smaller than the cell diameter. To mimic imperfections in the cell segmentation mask, we introduced normally distributed (std 80 nm) X–Y trajectory-to-trajectory shifts.

#### Simulation of video microscopy with SMeagol.

For simulation of fluorescent image stacks, single-molecule trajectories (on average three per cell) were generated from MesoRD trajectories via an experimentally derived point spread function and experimentally recorded fluorescence background. Simulated illumination time (1.5 ms) and frame time (20 ms) corresponded to in vivo microscopy experiments. The fluorophore brightness and bleaching time were tuned to match the in vivo experimental data. Cell segmentation masks generated by SMeagol (33) were filled to remove internal holes and then shrunken by 240 nm erosion.

#### Analysis of simulated data.

Simulated microscopy data were analyzed via the same image analysis and HMM pipelines as in vivo data, except that phase masks for cell segmentation were generated as described above.

## Supplementary Material

Supplementary File

Supplementary File

Supplementary File

## Data Availability

Experimental and simulated microscopy data generated and analyzed during the current study and the full DNA sequence are available in the SciLifeLab Data Repository (https://doi.org/10.17044/scilifelab.20502126) (53). The software used for microscopy simulations is publicly available at MesoRD (http://mesord.sourceforge.net/) and SMeagol (https://github.com/bmelinden/SMeagol_mat). The computational code used for analysis and plotting is available in the SciLifeLab Data Repository (https://doi.org/10.17044/scilifelab.20502126) (53). Video data have been deposited in SciLifeLab Data Repository (https://doi.org/10.17044/scilifelab.20502126) (53).
