# Supplementary material for: Spatiotemporal kinetics of the SRP pathway in live E. coli cells
Source: Proc Natl Acad Sci U S A. 2022 Sep 12;119(38):e2204038119. doi: 10.1073/pnas.2204038119 (PMC9499511; doi:10.1073/pnas.2204038119)
Supplement: Supplementary File [file pnas.2204038119.sapp.pdf]

Supplementary Information to:

Spatiotemporal kinetics of the SRP pathway in live *E. coli* cells

Ivan L. Volkov, Erik Lundin, Kalle Kipper, Mikhail Metelev, Spartak Zikrin, and Magnus Johansson

Department of Cell and Molecular Biology, Uppsala University

List of figures:

Figure S1 2
Figure S2 3
Figure S3 4
Figure S4 5
Figure S5 6
Figure S6 8
Figure S7 9
Figure S8 10
Figure S9 11
Figure S10 12
Figure S11 13
Figure S12 14
Figure S13 15
Figure S14 16
Figure S15 17
Figure S16 18
Figure S17 21
Figure S18 22
Figure S19 24
Figure S20 25
Figure S21 26

List of tables:

Table S1 6
Table S2 13
Table S3 19
Table S4 20
Table S5 23

Supplementary Notes:

Supplementary Note 1. Labelling of 4.5S RNA at the 3’ end is not expected to compromise the function and interactions of 4.5S RNA with other partners 2

Supplementary Note 2. Statistical criteria to define number of diffusion states 7

Supplementary Note 3. Estimation of fraction of membrane-associated molecules 12

Supplementary Note 4. Linking derived SRP cycle model with earlier SRP-specific ribosome profiling data 24

Supplementary Video Legend:

Supplementary Movie 1. Time lapse of LD655 labelled 4.5S RNA in live E. coli cells 27

Supplementary references 27

Supplementary Note 1. Labelling of 4.5S RNA at the 3’ end is not expected to compromise the function and interactions of 4.5S RNA with other partners

The 3’ end was considered to be an optimal position for 4.5S RNA labelling without compromising the functionality of 4.5S RNA *in vivo* for the following reasons:

1. The structure containing the most complete 4.5S RNA in complex with Ffh, FtsY, ribosome and translocon is shown in Fig. S1 [1]. The resolved structure is missing the last 7 nucleotides at the 3’ end due to the lower resolution in this region. However, as seen from the electron density map, the 3' end of 4.5S RNA does not form contacts with the other components and points away from all of the proteins and the ribosome. In other structures available from PDB [1,2], the 3’ end of 4.5S RNA is poorly resolved with a few tens of nucleotides in the 3’ end missing from the structure. The lack of electron density itself suggest that this part of the molecule is flexible and not involved in stable interactions with other partners.

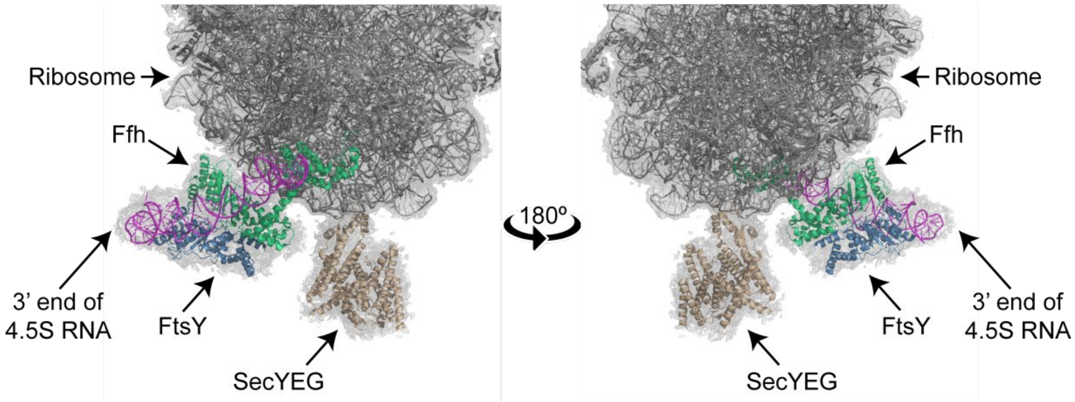

Figure S1. Quaternary complex between SRP (consisting of 4.5S RNA and Ffh), the SRP receptor (FtsY), and the translocon (SecYEG) bound to a translating ribosome, at 4.8 Å resolution [1], PDB 5NCO.

2. It has been shown that *E. coli* 4.5S RNA can be replaced by its homologs from other organisms, including the homolog from *Bacillus subtilis* which is twice larger than *E. coli* 4.5S RNA due to the large extension in the 3’ end [3].

3. It has been shown that expression of the *ffs* gene (i.e. 4.5S RNA) with missing terminator (resulting in longer-than-normal 4.5S RNA) is not toxic for *E. coli* [4].

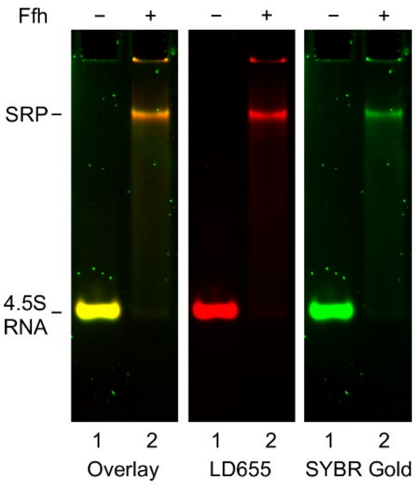

Figure S2. Gel mobility shift assay for LD655-labeled 4.5S RNA. In the presence of Ffh (3 µM to 1 µM RNA), no free RNA is observed.

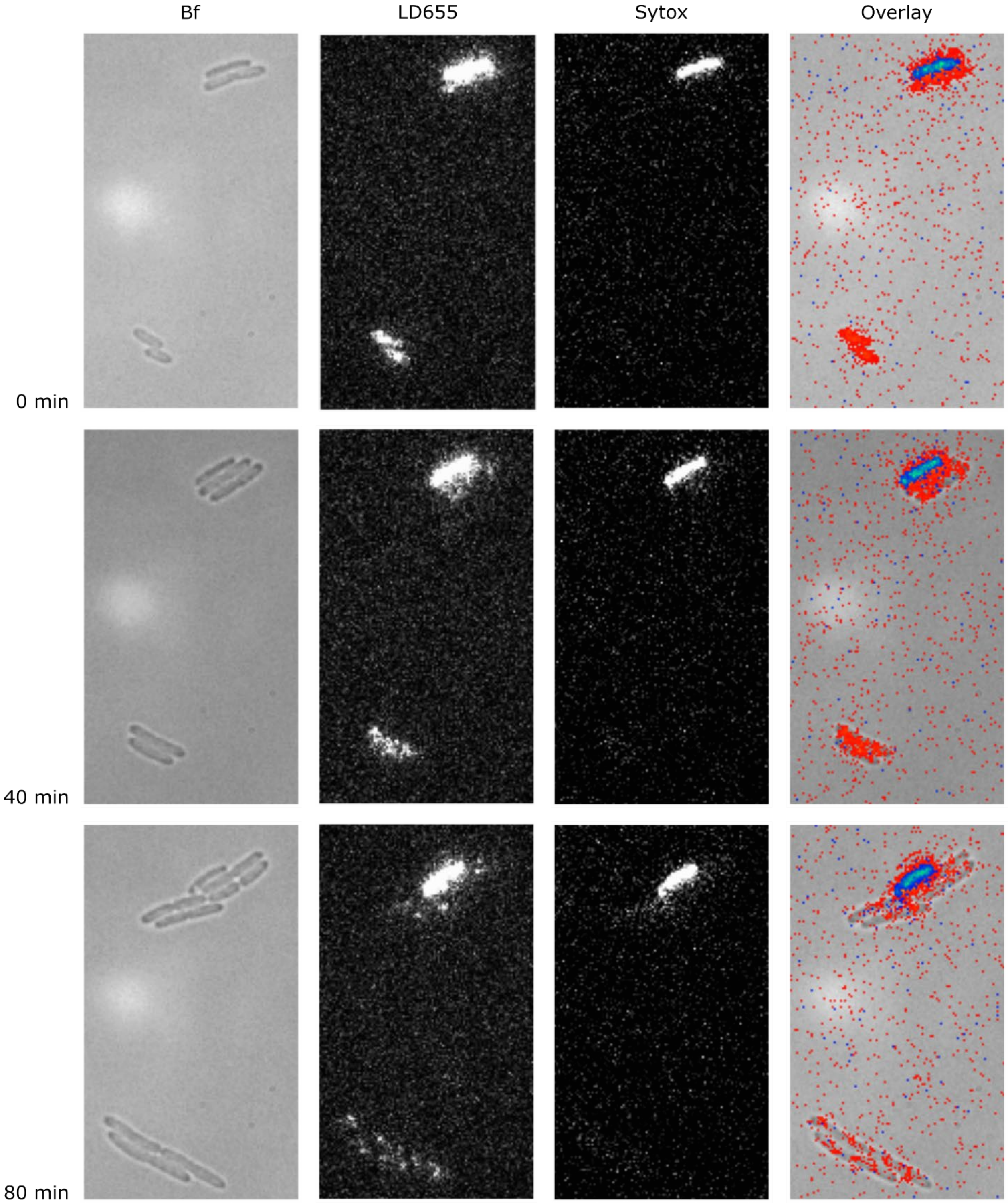

Figure S3. Bright field (Bf), LD655 fluorescence, and Sytox-blue fluorescence imaging of cells over time after electroporation with labeled 4.5S RNA. Labeled 4.5S RNA appear as bright pixels in the LD655 lane, and red pixels in the overlay lane. Sytox-blue stained, i.e. dead cells (bright pixels in Sytox lane, and blue pixels in overlay lane), were omitted in the analysis. Time zero corresponds to the beginning of microscopy sample incubation at 37° C, 1 hour after electroporation.

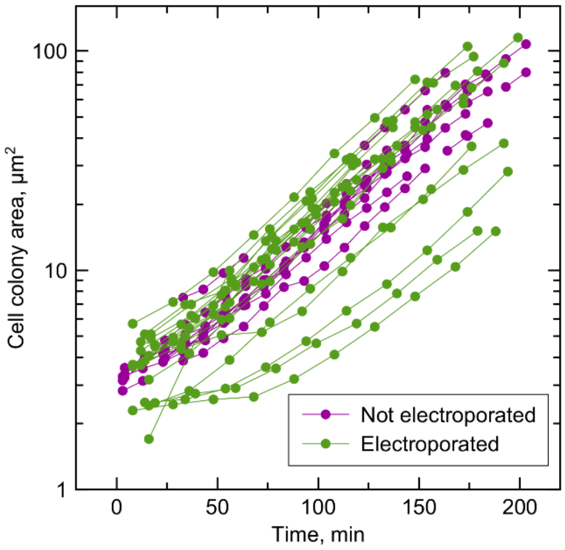

Figure S4. Cell growth followed under the conditions of a single-molecule tracking experiment. Electroporated and not electroporated cells treated equally, were sparsely placed on an agarose pad (time zero), and imaged every 10-20 min. 10-20 cell colonies of each type were selected, which, as in our experiments, fulfilled the following criteria: i) the cells grew and divided upon incubation (colony grown to ≥4 cells from a single cell), ii) displayed no Sytox-blue signal, and iii), contained internalized labeled 4.5S RNA (for the electroporated sample). Phase contrast images were used for segmentation of the cells, and the total colony area was computed. Growth curves were fitted with exponential curve within the time window where colonies were 4-16 cells in size. A doubling time of 37±4 min was found for electroporated cells, and 37±4 min for non-electroporated cells (mean and standard deviation from individual colonies). ~75% of the electroporated cells started to grow actively from time zero, while ~25% had a lag phase ~60 min, after which they began to grow with a rate undistinguishable from the control cells. Since for tracking experiments, only cell colonies containing 4-16 cells (thus actively growing) were selected, this data shows that by the time of data acquisition, cells are fully recovered after electroporation and grow as fast as non-electroporated cells.

Table S1. Comparative analysis of datasets obtained for electroporated 4.5S RNA labelled with LD655 and sulfo-Cy5 dyes in wt DH5α cells. Samples were prepared and imaged under identical conditions except that the sample with LD655 dye was supplemented with oxygen scavenging system components (see Materials and Methods).

	LD655	sulfo-Cy5	LD655 improvement over sufo-Cy5
Mean bleaching time of cell colony[1], frames	120 ± 50	24 ± 7	(5 ± 0.5) x
Number of cells in dataset	2969	4720	N/A
Number of trajectory steps in dataset	46896	33365	N/A
Mean trajectory length, frames	18	12	1.5 x
Mean number of trajectory steps per cell	15.8	7.1	2.2 x

[1] Intensity profiles were extracted from fluorescent image stacks using the ImageJ software and fitted with single-exponent decay in MagicPlot. Results from 9 randomly selected cell colonies per dataset were averaged. Error represents standard deviation from these 9 measurements.

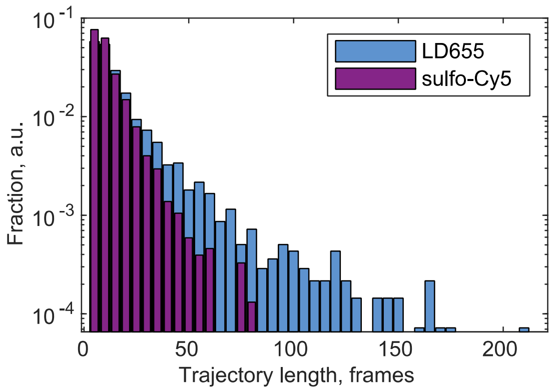

Figure S5.Trajectory length distribution for samples labeled with LD655 and sulfo-Cy5 dyes.

Supplementary Note 2. Statistical criteria to define number of diffusion states

To the best of our knowledge, there are no defined criteria for determination of exact number of diffusion states fully describing the mobility of biomolecules *in vivo*. Using simulated microscopy trajectories, representing molecules in discrete diffusion states in a confined cell volume, it has been shown that the commonly used algorithm vbSPT [5] overestimates the number of diffusion states, depending on the amount of data and complexity of the ground-truth model [6,7]. In addition, vbSPT cannot take into account dot localization uncertainties and motion blur, which is possible with our currently used algorithm. The Akaike information criterion (AIC), which is expected to predict the statistical quality of the model, also tend to overfit data [8].

Even if there existed a perfect statistical criterion predicting the model size, we would still, however, expect significant difficulties in determination of the number of diffusion states in an ensemble of living cells. Whereas the simulated microscopy data used in the aforementioned analyses were generated assuming Brownian diffusion in cells of same shapes and sizes with homogeneous viscosity, live-cell analyses of diffusing particles most probably include some cell-to-cell and in-cell spatial heterogeneity in diffusion due to, e.g., different location and number of nucleoids in cells at different stages of the cell cycle. Furthermore, in relation to SRP tracking in the present analysis (and tRNA tracking in our previous analyses [8]), we expect that the ribosome bound state would practically consist of a continuum of diffusion states, rather than a state described with defined diffusion coefficient, since elongating ribosomes can be bound alone on an mRNA or as polysomes with up to tens of ribosomes per mRNA. Hence, whereas the average diffusion coefficient is expected to be distinguishable between free 4.5S RNA, free SRP and ribosome bound SRP, the overall system is probably not described by a finite number of diffusion states, but rather by a number of diffusion ranges, more or less overlapping each other.

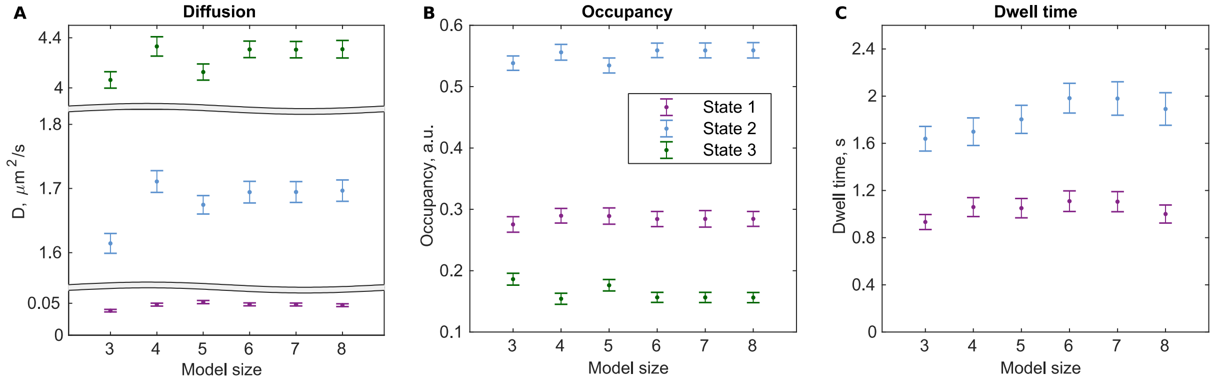

*Figure S6. Variation of HMM-fitted diffusion coefficient (**A**), occupancy (**B**) and dwell time (**C**) of coarse-grained states (thresholds 0.8 μm^2/s and 3 μm^2/s), depending on number of states in initial model for labeled 4.5S RNA in wt cells. Error bars represent boot strap-estimated errors. Variations in diffusion coefficients and occupancies of coarse-grained states do not exceed 10% in most of the cases, whereas variation of dwell times does not exceed 20% (except state 3).*

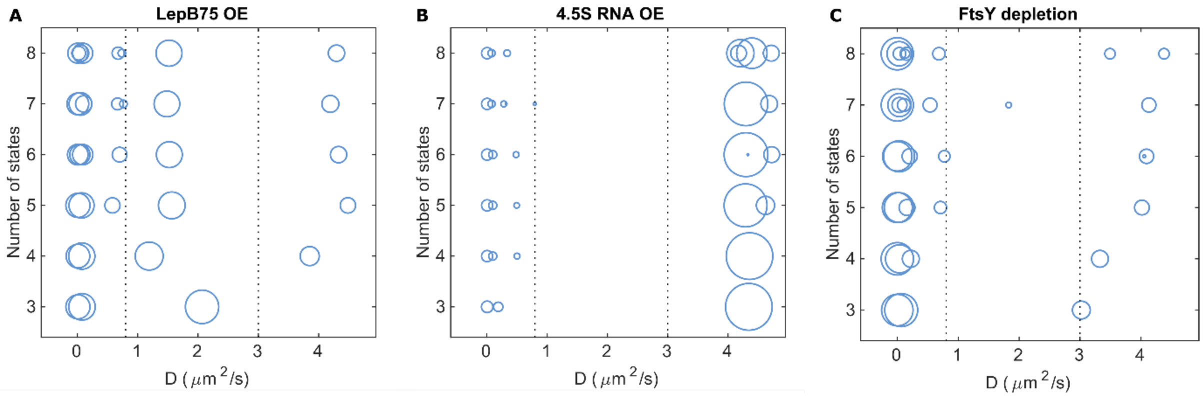

Figure S7. Distribution of HMM-fitted diffusion coefficients of labeled 4.5S RNA for different model sizes. ***A.*** *Cells over-expressing the LepB75 peptide.* ***B.*** *Cells over-expressing 4.5S RNA.* ***C.*** *FtsY depleted cells. The area of the circles is proportional to the occupancy of that particular state. Thresholds 0.8 μm²/s and 3 μm²/s used for coarse graining are shown with vertical dashed lines. Corresponding full models and coarse-grained results for all experiments are shown in Dataset 1.*

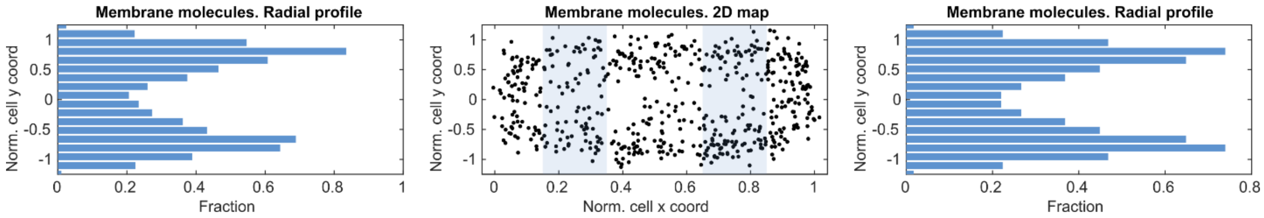

Figure S8. Spatial distribution of membrane-bound LacY-HaloTag labeled with the JF646-HaloTag ligand (state 1, 0.1 µm^2/s, >97% occupancy. The corresponding full model is shown in Dataset 1). In the middle panel, 600 randomly selected (out of 13911 total) dot locations are plotted on normalized cell coordinates, and regions used for projection to the short cell axis are highlighted in blue. The left panel shows the distribution of dot coordinates projected on the short cell radial axis (all 13911 data points are used to construct the histogram). The right panel shows the same data mirrored across the long cell axis to reduce experimental noise. Data from the right panel is used as membrane profile in Figures 2, 4, S13 and S15.

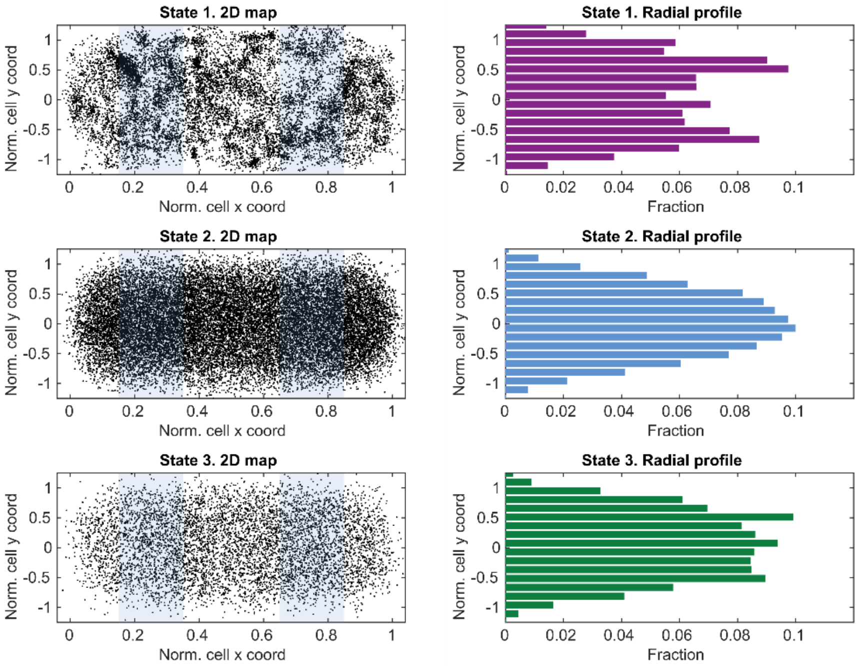

Figure S9. Spatial distribution of labelled 4.5S RNA in diffusion state 1 (ribosome-bound SRP), state 2 (free SRP), and state 3 (free 4.5S RNA). In the left panels, dot locations are plotted on normalized cell coordinates, and regions used for projection to the short cell axis are highlighted in blue. The right panels show the distribution of dot coordinates projected on the short cell radial axis. The data are the same as in Figure 2 (main text), but without mirroring across the long cell axis.

Supplementary Note 3. Estimation of fraction of membrane-associated molecules

We considered the steady-state total occupancy spatial distribution profile (T) of ribosome-bound state as a superposition of known "membrane" (M) and "cell interior" (C) profiles (Fig. S10) with unknown weight α:

$$T = \alpha M + (1-\alpha)C.$$

The membrane fraction α was obtained by least square fitting.

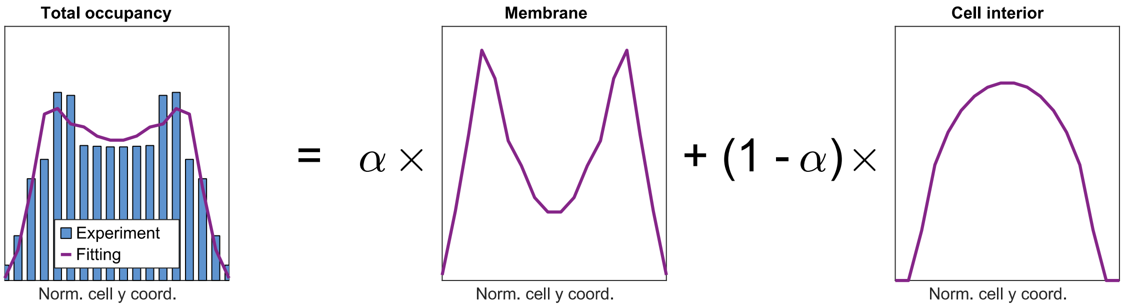

Figure S10. Estimation of membrane fraction of total occupancy of the ribosome-bound state (state 1). Fitting of experimentally derived profile by the sum of two profiles: "membrane" (LacY data, Fig S8) and "cell interior" (theoretical prediction for evenly distributed particles in a cylindrical cell without end caps).

The same procedure was performed to estimate respective fractions of "membrane" component (M) in SRP-ribosome binding events profile (B) and SRP release events profile (*R)*, i. e.:

$$R = \beta M + (1-\beta)C,$$
$$B = \gamma M + (1-\gamma)C.$$

The membrane fractions β and γ were obtained by least square fitting.

As α, β and γ vary slightly depending on the size of the initial HMM model, the number of bins of the profile histograms, and also the size of margins used to cut cell poles and the middle part of the cell while projecting coordinates to the short cell axis, we varied these parameters and followed values of α, β and γ (Fig. S11). Mean values of α, β and γ are presented in Table S2, with errors obtained either as standard deviation from the least square fitting procedure, or as a standard deviation of scattered α, β and γ values obtained with different profile construction parameters, depending on which error was largest.

The same procedure was applied to determine the apparent membrane fraction in total occupancy of state 2 (free SRP) and state 3 (free RNA) and results are shown in Table S2.

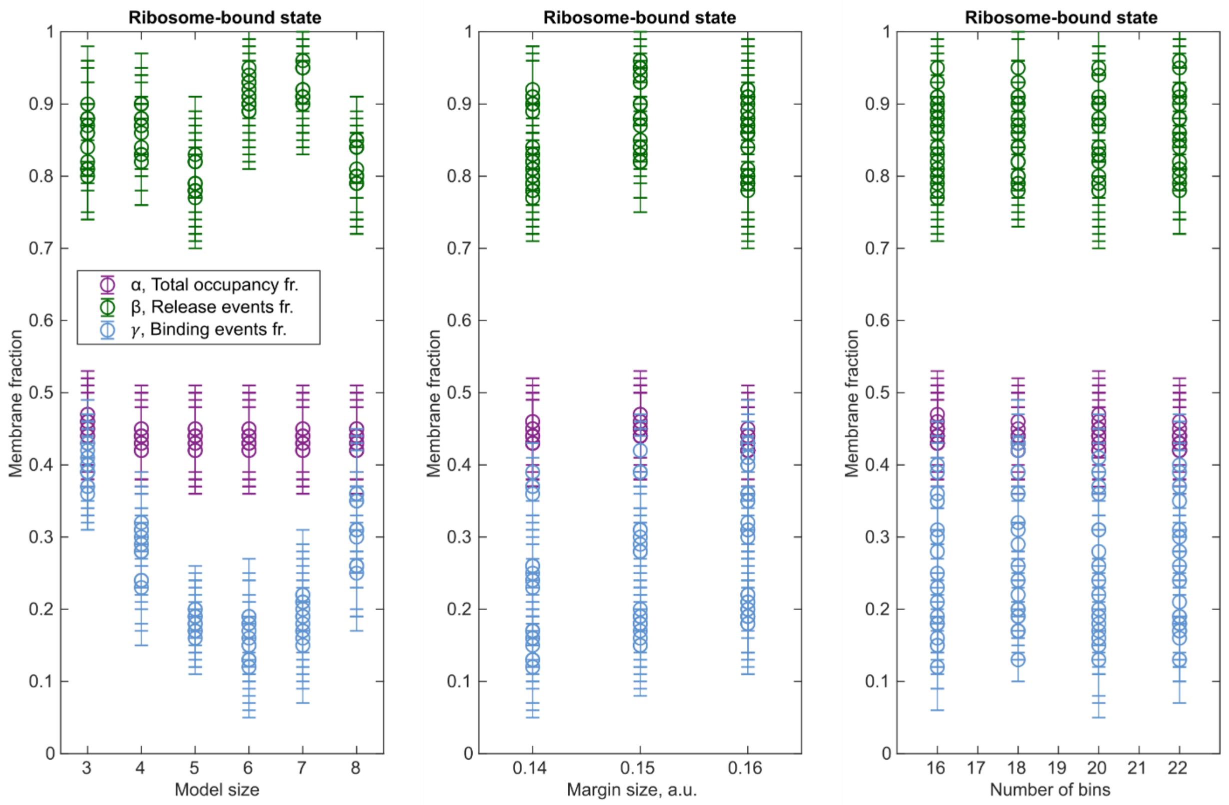

Figure S11. Variation of membrane fractions of total occupancy of state 1 (α), release events (β) and binding events (γ) from/to ribosome with changing HMM model size, margin size and number of bins. Error bars represent standard deviation obtained form least square fitting.

Table S2. Mean values of membrane fractions α, β and γ derived from least square fitting of experimental profiles as a sum of "membrane" and "cell interior" profiles.

Total occupancy of ribosome-bound state (state 1) , membrane fraction α	(44 ± 6)%
Release from ribosome, membrane fraction β	(86 ± 7)%
Binding to ribosome, membrane fraction γ	(25 ± 9)%
Total occupancy of free SRP state (state 2) , membrane fraction α	(0 ± 4)%
Total occupancy of free 4,5S RNA state (state 3) , membrane fraction α	(1 ± 3)%

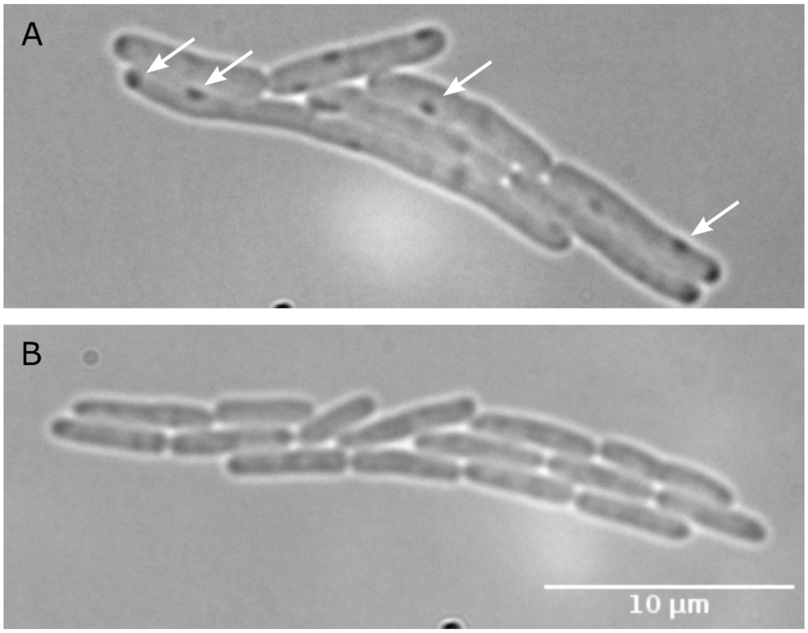

Figure S12. Bright field images of cell colonies formed from single cells approximately 4 hours after electroporation of labeled 4.5S RNA. ***A.*** *FtsY depleted cells (BW25113-Kan-AraCP-ftsY). Arrows indicate dark spots in the poles and by the membrane.* ***B****. DH5α cells (shown for comparison).*

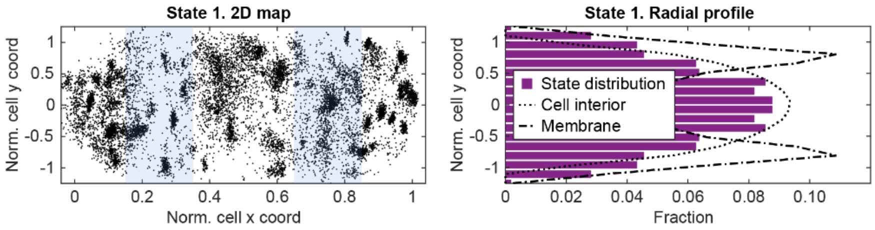

Figure S13. Spatial distribution of labelled 4.5S RNA in diffusion state 1 (ribosome-bound SRP) in FtsY depleted cells. In the left panel, dot locations are plotted on normalized cell coordinates, and regions used for projection to the short cell axis are highlighted in blue. The right panel shows the distribution of dot coordinates projected on the short cell radial axis. Data are mirrored across the long cell axis for better visibility. The dotted line corresponds to uniform distribution in the cytosol (theoretically predicted from the cell geometry), and the dash-dotted line to membrane distribution (obtained by tracking the inner membrane protein LacY, Fig. S8).

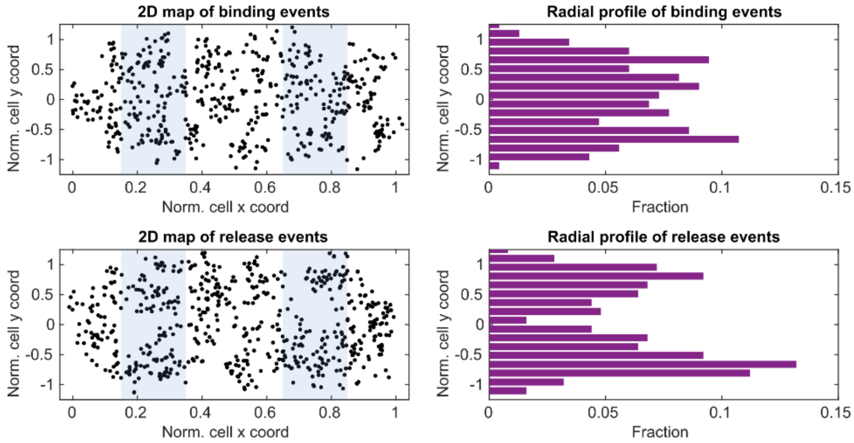

Figure S14. Spatial distribution of SRP-ribosome binding and release events detected as transitions to/from diffusion state 1 of labelled 4.5S RNA. In the left panels, locations of ribosome binding (upper) and release (lower) events are plotted on normalized cell coordinates. Regions used for projection to the short cell axis are highlighted in blue. The right panels show the distribution of binding and release events projected on the short cell radial axis. The data are the same as in Figure 4 (main text), but without mirroring across long cell axis. $N_{arrivals}$ = 184, $N_{departures}$ = 201. To increase precision, two positions of the particle following the binding event, and two positions of the particle preceding the release event were also included in the data, as explained in Materials and Methods.

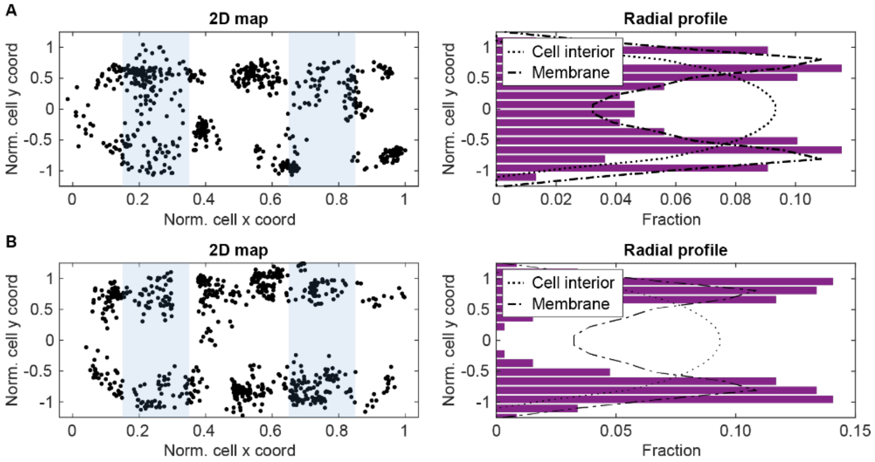

Figure S15. Spatial distribution of SRP-ribosome complexes after reaching the membrane proximity. In the left panels, single-molecule locations are plotted on normalized cell coordinates, and regions used for projection to the short cell axis are highlighted in blue. In the right panels, the distribution projected on the short cell radial axis is shown. Dotted lines correspond to uniform distributions in the cytosol (theoretically predicted from the cell geometry), and dash-dotted lines to membrane distribution (obtained by tracking the inner membrane protein LacY, Fig. S8). ***A****. Locations of complexes for which ribosome binding happened in cytosol, but plotted only from when they reached the membrane and further in time until SRP releases or the fluorophore is photobleached. As a rough approximation, events are attributed to happen in cytosol if the radial distance from the middle of the cell r < 0.5. Complexes are considered to have reached the membrane when r > 0.6.* ***B.*** *Locations of complexes after ribosome binding by the membrane and further in time until SRP releases or the fluorophore is photobleached. As a rough approximation events are attributed to happen by the membrane if the radial distance from the middle of the cell r > 0.6.*

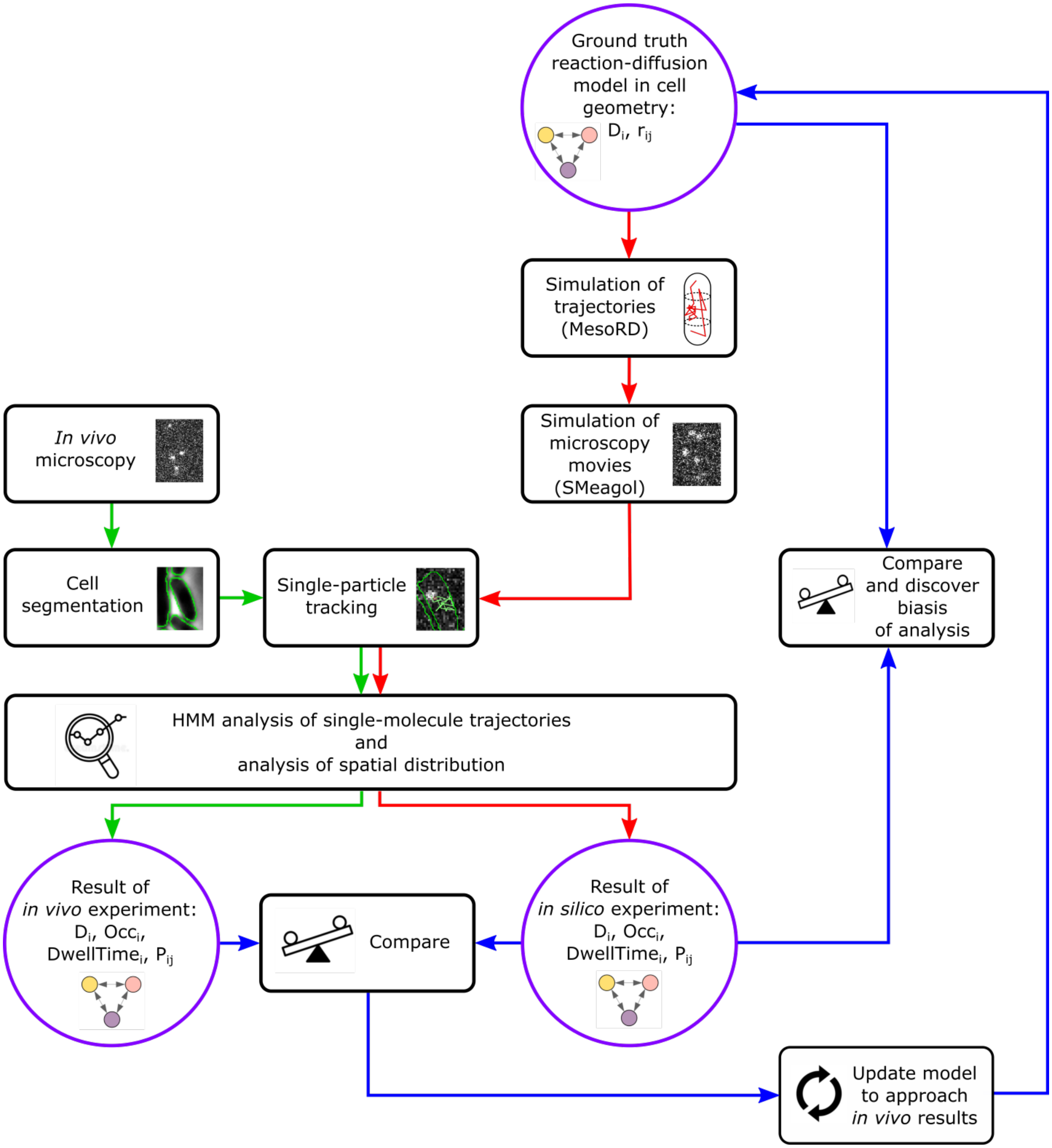

Figure S16. Simulation microscopy and data analysis workflow. D_i, Occ_i, $DwellTime_i$, – diffusion coefficient, occupancy and dwell time for the i:th diffusion state. r_{ij}, P_{ij} – reaction rate and transition probability from the i:th diffusion state to the j:th diffusion state. MesoRD [9] – software tool for stochastic simulation of reactions and diffusion. SMeagol [10] – software tool to simulate highly realistic microscopy data.

Table S3. Comparison of HMM-fitted in vivo microscopy data with HMM-fitted simulated microscopy data (8 states HMM models coarse grained to 3 states) and underlying simulated model ground truth with cytosol and membrane compartments. 3-8 states HMM models and coarse-grained results are shown in Dataset 1.

		State 1 (ribosome-bound SRP)			State 2 (SRP)	State 3 (4.5S RNA)
			Membrane	Cytosol		
Diffusion coefficient (µm²/s)	In vivo microscopy	0.047 +/- 0.002			1.70 +/- 0.02	4.3 +/- 0.1
	Simulated model ground truth	0.06			2.2	7.7
	Simulated microscopy	0.046 +/- 0.001			1.66 +/- 0.01	4.11 +/- 0.05
Occupancy (%)	In vivo microscopy	28+/-1	13+/-2	16+/-3	56+/-1	16+/-1
	Simulated model ground truth	26.8	17.2	9.6	57.9	15.3
	Simulated microscopy	28 +/- 1	13+/-1	15+/-1	57+/-1	15+/-1
Dwell time (ms)	In vivo microscopy	1000+/-80	440+/-70*	750+/-120*	1890+/-140	7000+/-14000
	Simulated model ground truth	740	479	342	1449	10000
	Simulated microscopy	1010+/-50	480+/-30*	710+/-60*	1700+/-100	9000+/-6000
Relative occupancy for compartments (%)	In vivo microscopy		44+/-6	56+/-6		
	Simulated model ground truth		64	35		
	Simulated microscopy		47+/-2	53+/-2		
Occurrence of arrival to compartments (%)	In vivo microscopy		25+/-9	75+/-9		
	Simulated model ground truth		23.5	76.5		
	Simulated microscopy		25+/-4	75+/-4		
Occurrence of departures from compartments (%)	In vivo microscopy		86+/-7	14+/-7		
	Simulated model ground truth		100	0		
	Simulated microscopy		90+/-4	10+/-4		

*Dwell times (τ) for membrane (1M) and cytosolic (1C) components of state 1 were computed based on steady-state assumption (i.e. equality of fluxes for certain parts of the cycle, see Fig. 5) from the following equations: $\frac{Occ_{1M}}{\tau_{1M}} = \frac{Occ_{state1}}{\tau_{state1}}$ and $\frac{Occ_{1C}}{\tau_{1C}} = OccurOfArr_{1C} * \frac{Occ_{state1}}{\tau_{state1}}$

Table S4. Comparison of HMM-fitted simulated microscopy data (8 states HMM model coarse grained to 3 states) with underlying ground-truth model consisting of only the cytosol compartment. 3-8 states HMM models and coarse-grained results are shown in Dataset 1.

		State 1 (ribosome-bound SRP)	State 2 (SRP)	State 3 (4.5S RNA)
Diffusion coefficient ($\mu m^2/s$)	Simulated model ground truth	0.06	2.2	7.7
	Simulated microscopy	0.054 +/- 0.001	1.68 +/- 0.01	4.16 +/- 0.06
Occupancy (%)	Simulated model ground truth	29.0	56.3	14.7
	Simulated microscopy	29 +/- 1	59 +/- 1	12 +/- 1
Dwell time (ms)	Simulated model ground truth	820*	1443	10000
	Simulated microscopy	1030 +/- 50*	1900 +/- 100	6700 +/- 2600

* In an attempt to find the source of the bias in dwell time estimation, a simpler model with one compartment was simulated. It resulted in smaller overestimation of dwell time of state 1 (~26%, compared to ~36% for two compartments model, Table S3).

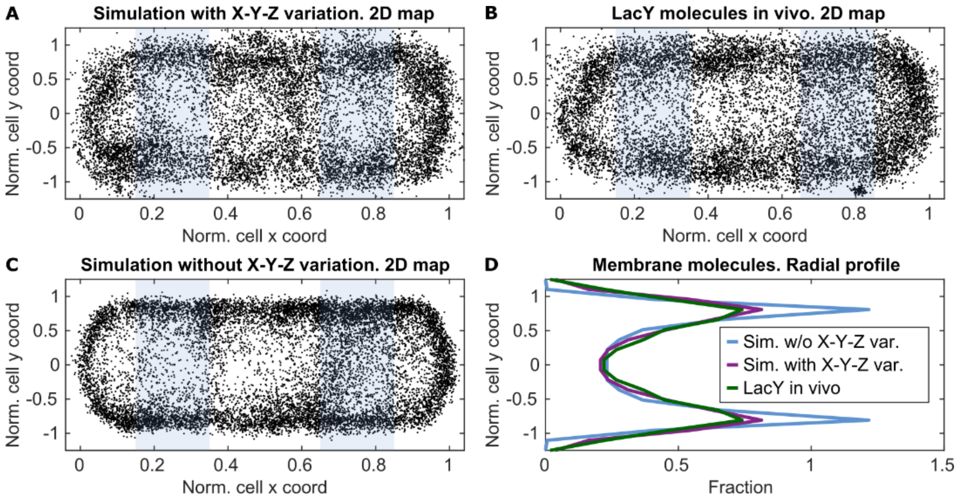

Figure S17. Spatial distribution of membrane molecules plotted on normalized cell coordinates. Regions used for projection to the short cell axis are highlighted in blue. Each scattered plot contains similar amount of data points which were randomly selected from total data. ***A****. Simulation of membrane molecule with X-Y and Z variation.* ***B.*** *In vivo single-particle tracking of labeled LacY protein (membrane benchmark, same data as in Fig. S8).* ***C.*** *Simulation of membrane molecule without X-Y and Z variation.* ***D.*** *Radial profiles of 2D spatial distributions.*

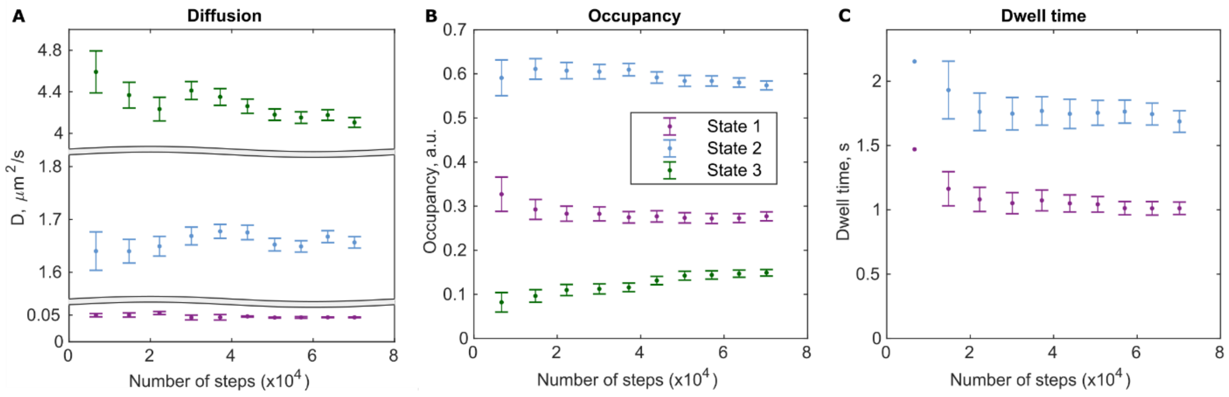

*Figure S18. Convergence of HMM-detected diffusion coefficient (**A**), occupancy (**B**) and dwell times (**C**) with increasing number of trajectory steps in tracking data for simulated two compartments SRP model (Figure S21a, Table S3).*

Table S5. Calculations of SRP cycling time in vivo based on estimated IMP and SRP copy numbers

Protein concentration[1] (M):	0.0050			
IMP concentration[2] (M):	0.0005			
Growth rate (dbl/h):	0.7	1.07	1.6	2.5
4.5S RNA concentration[3] (M):	$1.5 \cdot 10^{-6}$	$1.7 \cdot 10^{-6}$	$2.2 \cdot 10^{-6}$	$2.55 \cdot 10^{-6}$
SRP concentration[4] (M):	$3.7 \cdot 10^{-7}$	$4.3 \cdot 10^{-7}$	$5.5 \cdot 10^{-7}$	$6.38 \cdot 10^{-7}$
SRP cycling time[5] (s):	3.8	2.9	2.5	1.8

[1] ref [11]
[2] IMP around 10% of total protein content at exponential growth [12]
[3] ref [13]
[4] Assuming four 4.5S RNA per Ffh and SRP [14]
[5] Calculated as concentration of SRP multiplied by generation time divided by concentration of IMP.

Supplementary Note 4. Linking derived SRP cycle model with earlier SRP-specific ribosome profiling data

The distribution of dwell times of SRP in ribosome-bound states, obtained from the ground truth of the simulated model (Fig. S21a, Table S3), was converted from time units to number of codons translated during the SRP dwell using a translation rate of 17 aa/s [15,16] (Fig. S19, blue bars). The histogram shows a gradually increasing profile, resulting from the diffusion-limited time for membrane search, and a long "tail" representing the exponentially distributed membrane bound time built in to the model.

The data from SRP-specific ribosome profiling experiment [17] (*E. coli* MC4100 strain grown in RDM at 37 °C) represents the transcriptome-wide SRP interactome aligned to the position of initial SRP binding (Fig. 2a in [17]). Data from the published figure was digitalized using WebPlotDigitizer, background (mean translatome density) was subtracted, and the curve was normalized to the maximum value (Fig. S19, purple curve). The mean value of the SRP footprint from ribosome-profiling data was calculated to be 11 aa.

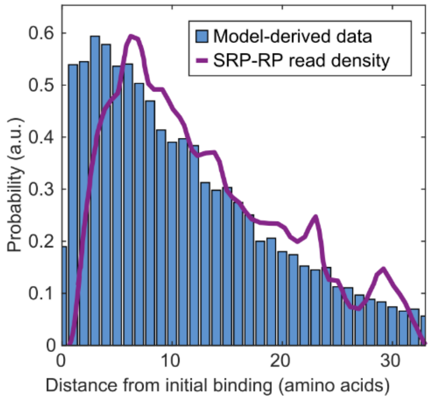

Figure S19. Comparison of dwell times for SRP in ribosome-bound state obtained from our simulated model, with SRP footprints obtained from SRP-specific ribosomal profiling (transcriptome-wide SRP interactome aligned to the position of initial SRP binding) [17].

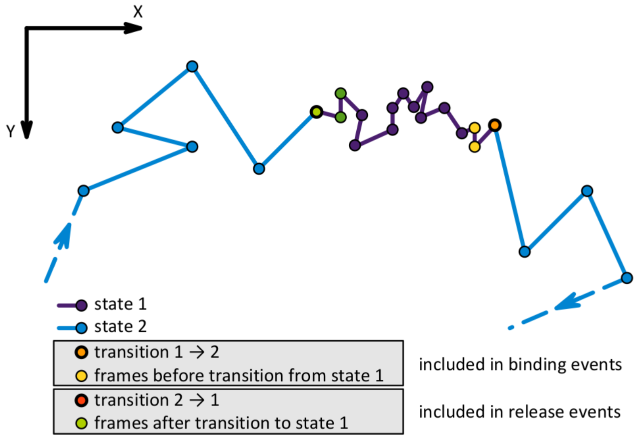

Figure S20. Schematic representation of a trajectory for particle experiencing transitions between state 2 → state 1 → state 2.

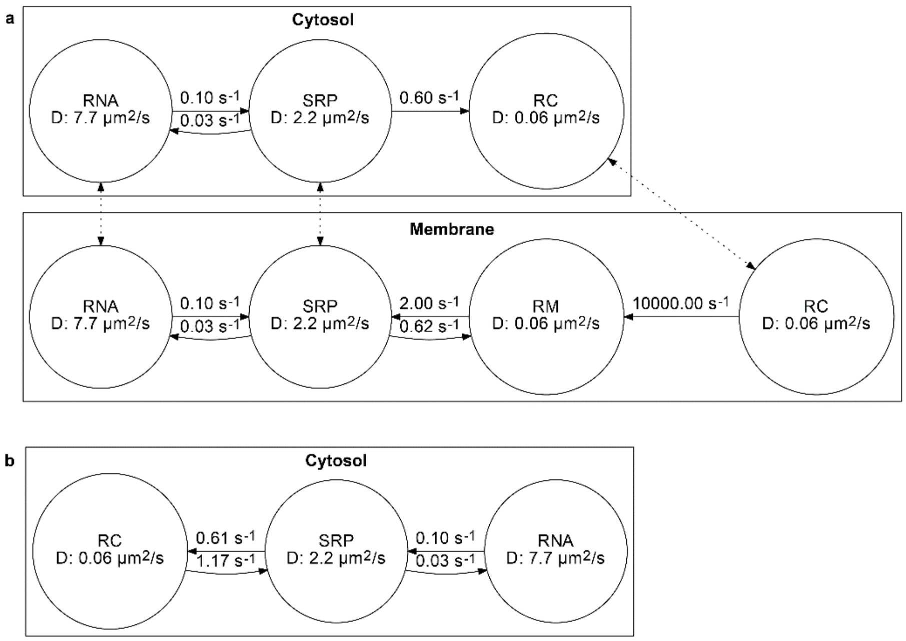

*Figure S21. 4.5S RNA reaction-diffusion model simulated in MesoRD (see Materials and Methods). Model including cytosol and membrane compartments (**a**), and model including only cytosol compartment (**b**). Resulting state occupancies and dwell times are shown in Table S3 and Table S4 respectively.*

Supplementary Video Legend

Supplementary Movie 1. Time lapse of LD655 labelled 4.5S RNA tracked at 20 ms per frame in live *E. coli* cells. The diffusion trajectory is color-coded with respect to HMM estimated diffusion state (coarse grained 3 states model). Playback speed is 10 frames per second (i.e. 5 times slower than reality).

Supplementary References

1. Jomaa, A. *et al.* Structure of the quaternary complex between SRP, SR, and translocon bound to the translating ribosome. *Nat. Commun.* **8**, (2017).
2. Jomaa, A., Boehringer, D., Leibundgut, M. & Ban, N. Structures of the E. coli translating ribosome with SRP and its receptor and with the translocon. *Nat. Commun. 2016 71* **7**, 1–9 (2016).
3. Struck, J. C., Lempicki, R. A., Toschka, H. Y., Erdmann, V. A. & Fournier, M. J. Escherichia coli 4.5S RNA gene function can be complemented by heterologous bacterial RNA genes. *J. Bacteriol.* **172**, 1284–1288 (1990).
4. Fukuda, S. *et al.* The Biogenesis of SRP RNA Is Modulated by an RNA Folding Intermediate Attained during Transcription. *Mol. Cell* **77**, 241-250.e8 (2020).
5. Persson, F., Lindén, M., Unoson, C. & Elf, J. Extracting intracellular diffusive states and transition rates from single-molecule tracking data. *Nat. Methods 2013 103* **10**, 265–269 (2013).
6. Vink, J. N. A., Brouns, S. J. J. & Hohlbein, J. Extracting Transition Rates in Particle Tracking Using Analytical Diffusion Distribution Analysis. *Biophys. J.* **119**, 1970–1983 (2020).
7. Lindén, M. & Elf, J. Variational Algorithms for Analyzing Noisy Multistate Diffusion Trajectories. *Biophys. J.* **115**, 276–282 (2018).
8. Volkov, I. L. *et al.* tRNA tracking for direct measurements of protein synthesis kinetics in live cells. *Nat Chem Biol* **14**, 618–626 (2018).
9. Hattne, J., Fange, D. & Elf, J. Stochastic reaction-diffusion simulation with MesoRD. *Bioinformatics* **21**, 2923–2924 (2005).
10. Lindén, M., Ćurić, V., Boucharin, A., Fange, D. & Elf, J. Simulated single molecule microscopy with SMeagol. *Bioinformatics* **32**, 2394–2395 (2016).
11. Milo, R. What is the total number of protein molecules per cell volume? A call to rethink some published values. *BioEssays* **35**, 1050–1055 (2013).
12. Schmidt, A. *et al.* The quantitative and condition-dependent Escherichia coli proteome. *Nat. Biotechnol.* **34**, 104–110 (2016).
13. Dong, H., Nilsson, L. & Kurland, C. G. Co-variation of tRNA abundance and codon usage in Escherichia coli at different growth rates. *J. Mol. Biol.* **260**, 649–663 (1996).
14. Jensen, C. G. & Pedersen, S. Concentrations of 4.5S RNA and Ffh protein in Escherichia coli: The stability of Ffh protein is dependent on the concentration of 4.5S RNA. *J. Bacteriol.* **176**, 7148–7154 (1994).
15. Young, R. & Bremer, H. Polypeptide chain elongation rate in Escherichia coli B/r as a function of growth rate. *Biochem. J.* **160**, 185–194 (1976).
16. Dai, X. *et al.* Reduction of translating ribosomes enables Escherichia coli to maintain elongation rates during slow growth. *Nat. Microbiol.* **2**, 1–9 (2016).
17. Schibich, D. *et al.* Global profiling of SRP interaction with nascent polypeptides. *Nature* **536**, 219–223 (2016).
